# Strategies to Improve Electrical Conductivity in Metal–Organic Frameworks: A Comparative Study

**DOI:** 10.1021/acs.cgd.3c01162

**Published:** 2024-02-26

**Authors:** Rajat Saha, Kajal Gupta, Carlos J. Gómez García

**Affiliations:** † Departamento de Química Inorgánica, Universidad de Valencia, C/Dr. Moliner 50, 46100 Burjasot, Valencia, Spain; ‡ Department of Chemistry, Nistarini College, Purulia, 723101, WB India

## Abstract

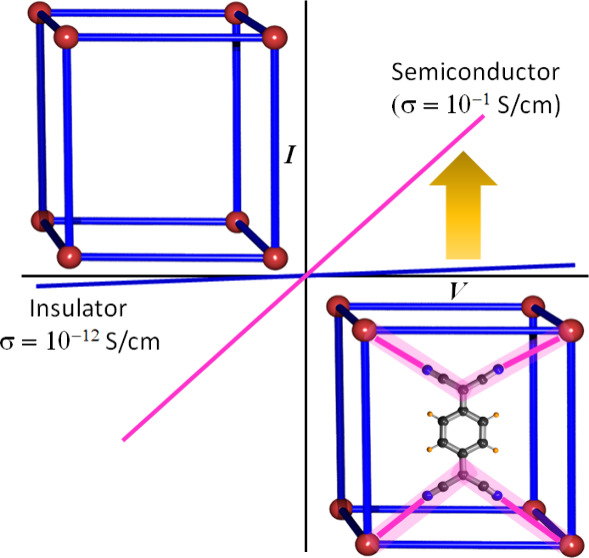

Metal–organic frameworks (MOFs), formed by the combination of both inorganic and organic components, have attracted special attention for their tunable porous structures, chemical and functional diversities, and enormous applications in gas storage, catalysis, sensing, etc. Recently, electronic applications of MOFs like electrocatalysis, supercapacitors, batteries, electrochemical sensing, etc., have become a major research topic in MOF chemistry. However, the low electrical conductivity of most MOFs represents a major handicap in the development of these emerging applications. To overcome these limitations, different strategies have been developed to enhance electrical conductivity of MOFs for their implementation in electronic devices. In this review, we outline all these strategies employed to increase the electronic conduction in both intrinsically (framework-modulated) and extrinsically (guests-modulated) conducting MOFs.

## Abstract 2

We present different strategies used to improve the electrical conductivity of MOFs: use of mixed valence metal ions, soft-donor ligands, extended conjugated organic ligands, incorporation of electroactive guest species, composite formation, etc. A brief overview on thin film fabrication and potential applications of MOFs are also presented together with the present challenges and future prospects.

## 1 Introduction

In modern society, the implications of electronic devices are enormous. Currently the main efforts are focused on the miniaturization and more efficient device fabrication at the nanoscale.^1,2^ Although classic metals, such as Cu, Ag, etc., are commonly used as conducting materials, there are also many applications that require semiconductors,^3^ including inorganic^4^ and organic ones.^5^ Pure elements (Si, Ge), metal-oxides/chalcogenides (ZnO, CdS, etc.), and doped metals (groups II–VI or III–V) have been used extensively in the semiconductor industry.^4,6−8^ The electron conduction in these inorganic materials follows the band model.^9^ On the other hand, organic molecules, charge transfer donor–acceptor molecules and organic polymers have gained much attention in organic electronics.^10^ Electron conduction in organic molecules and polymers depends on the conjugation of π-electrons, while the electron/hole transfer occurs between the donor–acceptor pairs.^11^ Thanks to their physical and chemical properties and their charge transport behavior, hybrid organic–inorganic structures as metal–organic frameworks (MOFs) have found a rapid development in the past decade as conducting materials.^12−14^

Metal–organic frameworks (MOFs) are a class of hybrid organic–inorganic materials having potential voids and a periodic three-dimensional structure formed by connected discrete metal ions or clusters with different di- or polytopic organic ligands.^15−18^ The most interesting feature of MOFs is that their structure, topology, and pore functionality can be tuned in a controlled manner through judicious selection of metal ions and bridging organic ligands.^19,20^ These distinct structural features of large porosity, chemically functionalized cavities, flexible skeletons, etc., make them suitable for gas and solvent adsorption,^21,22^ storage^23^ and separation,^24^ catalysis,^25^ sensing,^26^ drug delivery,^27^ etc. Recently, electrically conductive MOFs^28,29^ have gained much attention for their numerous applications in electrocatalysis,^30^ capacitors,^31^ charge storage,^32^ chem-resistive sensing,^33^ etc., along with their porosity-based functionalities. Generally, small organic ligands, as used in conventional MOF synthesis, are weak electrical conductors, and the poor overlap between p-orbitals of ligands and d-orbitals of metal ions results in insulating or poorly conducting MOFs, which constitutes the major obstacle for practical applications of conducting MOFs.^34^ However, conducting MOFs have several advantages, like: (a) the structural rigidity and the doping of inorganic semiconductors can be modified through organic functionalization; (b) amorphous organic polymers can be converted into crystalline MOF structures trough bonding with metal ions; (c) the infinite choice of metal ions and organic bridging ligands provides enormous opportunity to modulate the structure and topology, i.e., the compositional versatility offers the possibility to modulate the functionality through infinite ways; (d) detailed structural analysis offers a platform to tailor the electrical properties for suitable and desired applications; and (e) the combination of metal ions and organic ligands offers the possibility to explore multifunctionality like chem-resistive sensors based on porosity and conductivity, magneto-resistive devices based on conducting and magnetic MOFs, etc.

In 2000, Coronado and Gómez-García et al. reported the electrical properties of a host–guest MOF formed by the encapsulation of BEDT-TTF within the interlamellar space of a 2D magnetically ordered framework.^35^ Four-probe electrical conductivity measurements within the plane showed a very high room temperature conductivity value of ∼250 S cm^–1^ and metallic conductivity within the temperature range of 2–300 K. The material also shows ferromagnetic long-range order below 5.5 K with the presence of magnetic hysteresis at 2 K. Application of a magnetic field perpendicular to the layers shows negative magnetoresistance below 10 K. They have also modified the guest species to design different conducting host–guest system.^36,37^ In 2009, Takaishi et al. synthesized another electrically conducting MOF, Cu[Cu(pdt)_2_] (pdt = 2,3-pyrazinedithiolate), using a thiol-based organic ligand. This mixed-valence [Cu^I^Cu^III^] framework shows a high electrical conductivity of about 6 × 10^–4^ S cm^–1^ at 300 K.^38^ In 2010, Kobayashi and co-workers reported a similar MOF, Cu[Ni(pdt)_2_], with a related dithiol-based organic moiety showing an electrical conductivity of 10^–8^ S cm^–1^.^39^ Since then, many researchers have focused on this topic in order to design conducting MOFs with high conductivities and current densities, which are required for practical applications of conducting MOFs. Improvement of electrical conductivity in MOFs, i.e., design of highly conducting MOFs, is a big challenge. The proper selection through crystal engineering of metal ions and organic ligands is very important in order to obtain high charge mobilities in MOFs.

**Figure 1:**
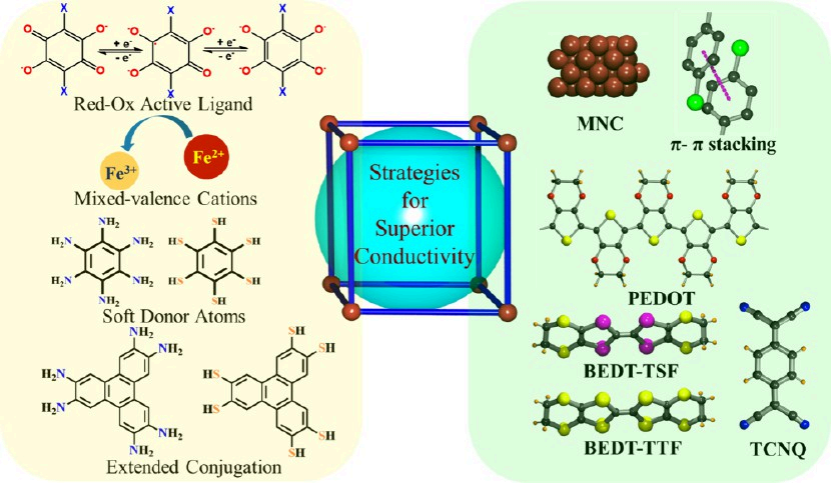
Schematic diagram for designing and improving electrical conductivity in both intrinsically (left side) and extrinsically (right side) conducting MOFs. Color code: C = gray, S = yellow, Se = pink, N = blue, O = red, Cl = green.

Depending on the charge transport pathway, these conducting MOFs can be classified into two different categories: (a) *intrinsically conducting MOFs*, when the charge transport occurs only through the metal–ligand backbone of the framework, and (b) *extrinsically conducting MOFs*, when the charge transport occurs through the guest species within the host framework.^34,40^ To date, some review articles, such as those of Zamora,^12^ Dinca,^28,34^ Alledorf,^41^ etc., have comprehensively described the progress in this field, whereas Morris,^42^ Pang,^43^ and Zhu^44^ have highlighted the design principles in conducting MOFs. Nevertheless, to date, there is no review article focusing on the strategies to modulate the charge transfer pathway in order to increase the electrical conductivity in MOFs and CPs. Here we show the strategies used by different groups to design both intrinsically and extrinsically conducting MOFs with high electrical conductivity. We discuss the band and hopping charge transport mechanisms operative in MOFs, and show the different charge transport pathways: (a) through- bond, (b) through- layer, (c) through- space, (d) through- guest, and (e) redox hopping in MOFs. We show that for intrinsically conducting MOFs, the charge transfer can be improved by using (i) ligands with soft donor atoms, (ii) redox-active noninnocent ligands, (iii) ligands with extended π-conjugated skeletons, (iv) mixed-valence metal centers, and (v) electron-rich metals (low-valent and/or groups 8 to 12). On the other hand, the electrical conductivity of extrinsically conductive MOFs can be tuned by incorporating (a) metal ions and metal nanoclusters, (b) different types of organic, inorganic, and organometallic molecules, and (c) organic conducting polymers (Figure 1).

### 1.1 Conductivity Measurements

The electrical conductivity (σ) of a conductor can be expressed as σ = *ne*μ where *n*, *e*, and μ are the concentration, electronic charge, and mobility of the carriers, respectively. The carrier concentration decreases exponentially as the band gap increases. A way to increase the conductivity is, therefore, by oxidation or reduction, since both processes may raise the carrier concentration by increasing the electron population or decreasing the band gap. Doping of metals may lead to further supply of potential carriers, but in order to contribute to conductivity, these carriers should be mobile. Carrier mobility depends on the type of transport mechanism and pathway (interchain, single-chain, interparticle transport, etc.). The conduction mechanisms can be grouped into three models: variable range hopping model (VRH), tunnelling model, and superlocalization model (see below).

**Figure 2:**
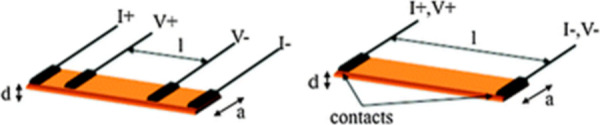
Schematic diagram of four-probe (left) and two-probe (right) methods for conductivity measurements. Dimensions l, a, and d are the voltage probes distance, the sample width, and sample thickness, respectively. Reproduced with permission from ref (12). Copyright 2012 Royal Society of Chemistry.

**Figure 3:**
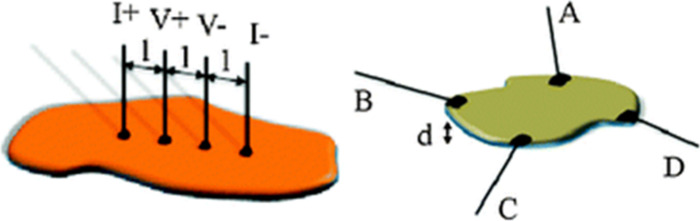
The four points (left) and van der Pauw (right) methods used for measuring electrical conductivity in very thin samples. Reproduced with permission from ref (12). Copyright 2012 Royal Society of Chemistry.

The resistance of a sample can be measured by (a) four-probe and (b) two-probe DC techniques. The four-probe method (Figure 2) can be used to measure the resistivity of any material, either bulk or thin film. The four-probe setup consists of four wires (usually tempered Cu, Pt, or Au wires of 25–100 μm diameter) connected to the sample by means of cold welding made with gold, silver, or graphite pastes (emulsions of fine powders dispersed in an organic solvent). A particular case is the four points technique, which is used when the samples are thin but firm. It uses four equidistant contacts (usually tungsten metal tips) connected by simply touching the sample (Figure 3). A high impedance current source is used to supply current through the outer two probes, and a voltmeter measures the voltage across the inner two probes to determine the sample resistivity. The typical current used ranges from 2 nA to 100 mA as in this region no Joule heating effect is observed.

The van der Pauw method (Figure 3) is used for irregular and very thin samples. In this method, the four wires are connected to points of the periphery of the thin sample, forming an approximate square. Initially, the current is applied though two consecutive points (AB), and the voltage is measured across the other two points (CD) to obtain R_CD_. The resistance in the other direction, R_AD_, is measured in a similar way, applying the current through BC and measuring the voltage across AD. The resistivity of the sample can be calculated from the R_AD_ and R_CD_ values.^12^ The two-probe method is used for highly resistive samples, where the contact resistance is negligible compared to the sample resistance.

### 1.2 Conducting Mechanisms

**Figure 4:**
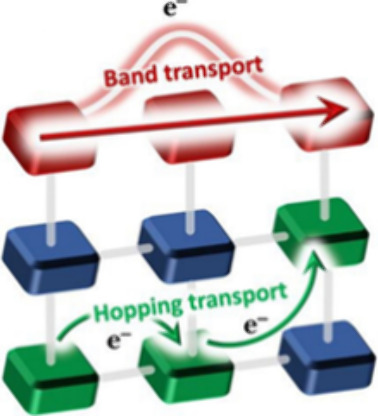
Two most probable charge-transport mechanisms applicable for MOFs. Reproduced with permission from ref (45). Copyright 2019 Royal Society of Chemistry.

Two different types of charge transport mechanisms are operative in solid materials: band-like charge transport and redox hopping (Figure 4).^45^ For most MOFs, the covalent metal–ligand bond formed by strong overlap between the metal ions and donor ligands orbitals, form continuous conduction bands. The energy gap (*E*_g_) between the conduction and valence bands determines the electrical properties of the materials that can be classified as metallic conductors, semiconductors, and insulators. In insulators, the band gap is above ≈3.6 eV, and in semiconductors it is below ≈3.6 eV,^46^ whereas in metallic conductors, the conduction and valence bands merge, with the Fermi level crossing this band. With increasing temperature, the electrical conductivity decreases for metallic conductors, while it increases for semiconductors. In metallic conductors at high temperatures, the electron–phonon coupling reduces the electron mobility and, accordingly, the electrical conductivity. For semiconductors, the increase in temperature increases the number of charge carriers in the conduction band, resulting in an increase of the conductivity. The electrical conductivity of classical semiconductors follows the Arrhenius equation: σ = σ_0_ exp −(*E*_a_/*k*T), where *E*_a_ is the activation energy, corresponding to half of the band gap (*E*_a_ = *E*_g_/2).

Besides the delocalized model based on electron bands, there exists a localized model where the charges move through a so-called hopping mechanism, where charges “jump” through a phonon-assisted quantum tunnelling mechanism. In this hopping model, the conductivity is described by a general equation: σ = σ_0_ exp [(−T_0_/T)^α^], where the exponent α depends on the dimensionality (*d*) of the conducting lattice in the form: α = 1/(1 + *d*). For three-dimensional materials, α = 1/4 and the expression becomes the Mott model,^47^ where the electron transport is similar to a self-exchange process between redox couples to maintain electroneutrality. In the hopping model, an increase of the temperature leads to an increase of the electron delocalization and, therefore, to an increase of the electrical conductivity.

According to Mott’s variable range hopping (VRH) theory, the conductivity is given by
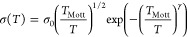
1
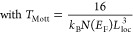
2where the VRH exponent (γ) is determined by the dimensionality of the conducting pathway:  and for three-, two-, and one-dimensional systems, γ becomes 1/4, 1/3, and 1/2, respectively. σ_0_ denotes the conductivity at infinite temperature, *T*_Mott_ is the Mott characteristic temperature, *k*_B_ is the Boltzmann constant, *N*(*E*_F_) is the density of states at Fermi level, and *L*_loc_ is the localization length. A sample with a 3D/2D/1D charge transport shows a linear dependence in a plot of ln [*T*^1/2^σ(*T*)] vs. *T*^–γ^ (with γ = 1/4, 1/3, and 1/2 for 3D, 2D, and 1D, respectively). From the slopes of these plot, the *T*_Mott_ can be determined.

Most of the MOFs are either insulators or semiconductors. Nevertheless, there is an increasing number of MOFs presenting metallic conductivity.^48^ In most cases, the band model is used to explain their electrical conductivity, although in a few cases, the hopping mechanism has also been claimed.^49^

### 1.3 Conduction Pathways

**Figure 5:**
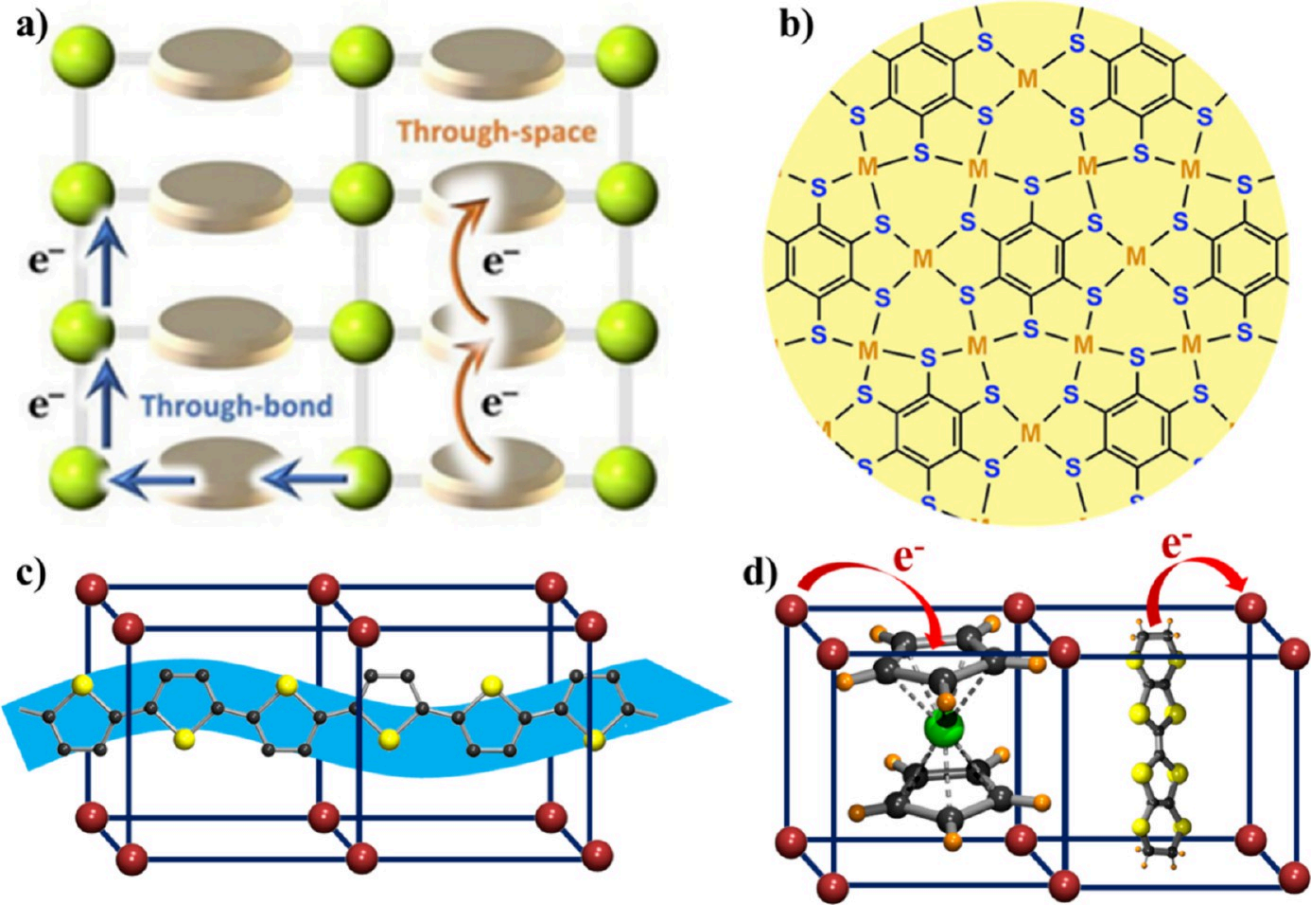
(a) Two different charge-transport pathways, through- bond and through- space, applicable for MOFs. Reproduced with permission from ref (45). Copyright 2019 Royal Society of Chemistry. (b) Through- plane charge transport based on metal–ligand d−π orbital overlap. Reproduced from ref (92). Copyright 2018 American Chemical Society. (c) Conductive polymeric guest-mediated charge transport within porous MOF and (d) two possible redox hopping mechanisms (lattice-to-guest or guest-to-lattice) for charge transport. Color code: C = gray, S = yellow, N = blue, O = red, and Fe = green.

Following the above-mentioned mechanisms, the charge transport in MOFs may occur through different pathways such as (a) through-bond, (b) through-layer, (c) through-space, (d) redox hopping, and (e) through-guest (Figure 5).

(a) **Through-bond.** MOFs containing (−M–X−)_*n*_ lattices (where X is the donor atom of the functional group of the ligand) can show such through-bond electron transport.^50^ Such transport is independent of the ligand backbone. The overlap between the metal ion (M) and donor atoms of the ligand (X) is the key factor for electron transport though this type of pathway. The use of soft donor atoms (S, P, Se, etc.) combined with transition metal ions can promote conductivity through such pathways, as observed in several MOFs showing (−M–X−)_*n*_ chains.^51^
(b) **Through-plane.** The use of organic ligands having extended conjugated organic cores, such as benzene, triphenylene, etc., and different coordinating groups, such as hydroxido, amine, diol, dithiol, etc.)^52−54^ can give rise to graphene-like 2D metal–organic sheets when bonded to transition metal ions. The charge transport of this type of 2D metal–organic layers is dependent on both the metal–ligand overlap as well as on the extended in-plane conjugation though the ligand. The extended π–d conjugation is the most operative charge transport pathway for such type of 2D metal–ligand layers.^55^ As expected, the interlayer π–π interaction is very important for the charge transport perpendicular to the plane (through- space).
(c) **Through-space.** In organic electronics, intermolecular π-interactions are used to increase the dimensionality of electrical conductors and semiconductors.^56^ Similarly, 1D or 2D MOFs containing aromatic rings at their ligand backbones can also participate in different interchain or interlayer weak interactions to form 3D supramolecular structures. These interchain or interlayer π-interactions have been used by different research groups as the charge transport pathway.^57,58^ The charge carrier efficiency is dependent on the strength of the π-interactions, which depend on the distance and geometry of the metal–organic moieties. In 1D MOFs, both the through-bond and through-space charge transport pathways can act simultaneously, and in some cases, it is difficult to predict the contribution of each of them. A similar case occurs in 2D MOFs, where both through-plane and through-space transport pathways are operative.
(d) **Redox Hopping.** In the absence of any particular charge transport pathway, electron transport may occur through a hopping process.^59,60^ This mechanism is operative when the metal–ligand overlap is very poor or its nature is mostly ionic. Under these circumstances, the localized electrons jump between different redox active sites. Therefore, the presence of redox-active metals or linkers is needed to promote such charge hopping.
(e) **Through-guest.** Taking advantage of the void space present within the MOFs, several research groups have incorporated different types of electroactive guest species: metal ions, metal nanoclusters, organic and inorganic molecules, redox-active molecules, polymers, etc., to design conducting MOFs and materials.^61,62^ Two different approaches have been used to develop such conducting guest@MOFs materials: (i) templated synthesis and (ii) postsynthetic incorporation. The incorporated guest molecules form charge transport pathways within the material either through host–guest or guest–guest interactions. In the case of organic conducting polymers, the charge transport pathway is provided by the continuous conjugated bond network of the polymer.

In all these conducting pathways, the dimensionality of the MOFs is expected to play a key role in determining not only the electrical conductivity but also the conducting pathway. The study of the role of the dimensionality on the electrical conductivity and its mechanism would require the preparation of polymorphic conducting MOFs only differing in their dimensionalities. As far as we know, there are no published examples of conducting MOFs with the same composition (metal ions and ligands) but different dimensionality.

## 2 Design Strategies to Improve Charge Transport in MOFs

In the past decade, the number of conducting MOFs has experienced a remarkable increase. Based on the charge transport pathway, conducting MOFs are classified into intrinsically and extrinsically conducting MOFs. Different pre-synthetic and post-synthetic strategies have been developed to obtain high conductivity in MOFs.

### 2.1 Intrinsically Conducting MOFs

The electrical conductivity of the intrinsically conducting MOFs is an inherent property and thus can be modulated through the modification of both metal ions and organic ligands in the MOF. Several strategies have been developed in order to enhance the conductivity of intrinsically conducting MOFs.

#### 2.1.1 Incorporation of Redox-Active Ligands

**Figure 6:**
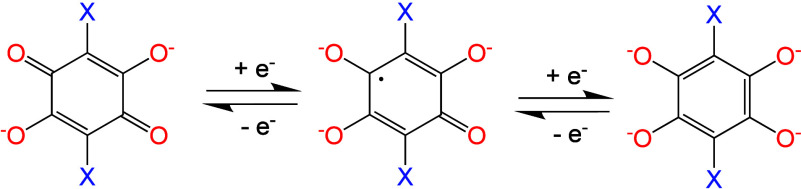
Different redox states (−2, −3, and −4) of the C_6_O_4_X_2_^*n*–^ moieties.

Organic bridging ligands having a conjugated organic core with variable oxidation states have gained significant attention in the design of conducting molecules and MOFs.^63^ Significant attention has been paid to those ligands having a catechol moiety (dihydroxybenzene, chloranilic acid, etc.)^64,65^ since they present several stable and easily interconvertible oxidation states through protonation and deprotonation. During the synthesis, the ligands can be easily reduced or oxidized to form redox-active frameworks with different charges or protonation degree. When these redox-active ligands form extended networks with extended conjugation, these frameworks may be highly conducting thanks to the electron hopping between the metal ion and the redox-active ligand (usually due to valence tautomerism). On the other hand, the electron conduction in MOFs is also dependent on the metal–ligand frontier orbitals overlap. The presence of an open shell in the outer orbital of both, the metal ion and ligand, may lead to optimal overlap that facilitates the electron conduction throughout the framework. One of the most studied redox-active ligands is 2,5-dihydroxybenzoquinone (H_2_dhbq, Figure 6, X = H) that has frontier orbitals with similar energies to those of many transition metals. This ligand may show up to three different valence states in metal–organic motifs (Figure 6). This valence tautomerism may result in an increase of the electrical properties and in a modulation of other properties. In this section, we discuss the conducting MOFs formed by using mixed-valence tetraoxolene-based ligands such as H_2_dhbq and the derivatives with X = Cl and Br, known as chloranilate (C_6_O_4_Cl_2_)^2–^ and bromanilate (C_6_O_4_Br_2_)^2–^, respectively (Figure 6).

Darago et al. have synthesized both the oxidized and reduced complexes of dhbq^*n*–^ and Fe^III^ with formulas (NBu_4_)_2_[Fe^III^_2_(dhbq)_3_] (**1-ox**) and Na_0.9_(NBu_4_)_1.8_[Fe^III^_2_(dhbq)_3_] (**1-red**) through selective reduction with Na of the oxidized compound (Table 1).^66^ Two-probe DC conductivity measurements on pressed pellets of the oxidized and reduced forms indicate that the oxidized compound is a better conductor than its reduced counterpart. The conductivity values are 0.16 and 0.0062 S cm^–1^ at 298 K, respectively. The authors demonstrated that the population of dhbq^2–^ vacancies increases due to reduction, and, as a result, unpaired electrons of dhbq^3–^ linkers hop to nearest neighbor dhbq^2–^ vacancies. The carrier mobility decreases, and, therefore, the conductivity also decreases. Both samples are semiconductors in the temperature range 70–300 K. Activation energies of the samples obtained from Arrhenius fit are 110 and 180 meV for **1-ox** and **1-red**, respectively. Fractional oxidation of **1-ox** may result in higher conductivity, but clean synthetic conditions for the oxidative deinsertion of the tetrabutylammonium cations have not yet been identified. These examples show that the ligand mixed-valence can serve as a highly efficient transport pathway within the MOF and that transition metal ions and semiquinoid ligands are promising scaffolds for delocalized and tunable electronic structures in MOFs.

**Table 1:** Conducting MOFs Prepared with Redox-Active Ligands

MOF	Formula	Dim	σ (S cm^–1^)	*E*_a_ (meV)	ref
**1-ox**	(NBu_4_)_2_[Fe^III^_2_(dhbq)_3_]	3D	0.16^a^	110	(66)
**1-red**	Na_0.9_(NBu_4_)_1.8_[Fe^III^_2_(dhbq)_3_]	3D	0.0062^a^	180	
**2-ox**	(Me_2_NH_2_)_2_[Fe_2_(C_6_O_4_Cl_2_)_3_]·2H_2_O·6DMF	2D	1.4 × 10^–2^^a^	260	(64)
**2-red**	(Cp_2_Co)_1.43_(Me_2_NH_2_)_1.57_[Fe_2_(C_6_O_4_Cl_2_)_3_]·4.9DMF	2D	5.1 × 10^–4^^a^	340	
**3-ox**	(Me_4_N)_2_[Mn_2_(C_6_O_4_Cl_2_)_3_]	2D	1.14 × 10^–13^^a^	740	(67)
**3-red**	Na_3_(Me_4_N)_2_[Mn_2_(C_6_O_4_Cl_2_^3–•^)_3_]·3.9THF	2D	2.27 × 10^–8^^a^	489	
**3-reox**	Na(Me_4_N)[Mn_2_(C_6_O_4_Cl_2_)_3_]·5.5THF·0.8CH_3_CN	2D	1.45 × 10^–13^^a^	–	
**4**	[Cr^III^Cl_2_(pyz)^•–^]_*n*_	2D	0.032^a^	–	(69)
**5**	[V^II^Cl_2_(pyz)]_*n*_	2D	10^–10^^a^	–	(70)
**6**	[Ti^III^Cl_2_(pyz)^•–^]_*n*_	2D	5.3^a^	–	(70)

a Two-probe on pressed pellets.

**Figure 7:**
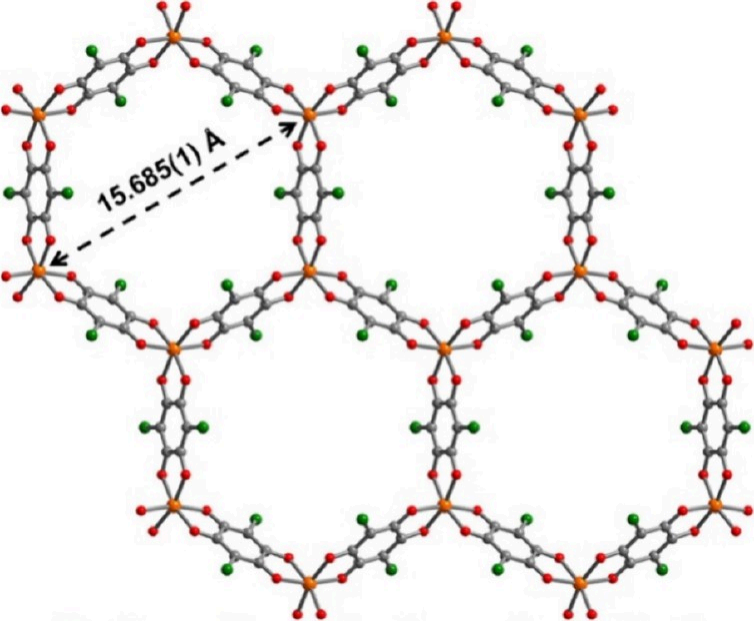
X-ray crystal structure of the [Fe_2_(C_6_O_4_Cl_2_)_3_]^3–^ layer as observed in **2-red**, viewed perpendicular to the anionic layer. Cations and DMF molecules are omitted for clarity. Color code: Fe = orange, Cl = green, O = red, and C = gray. Reproduced from ref (64). Copyright 2017 American Chemical Society.

DeGayner and co-workers reported the electrical conductivities of the two-dimensional MOF (Me_2_NH_2_)_2_[Fe_2_(C_6_O_4_Cl_2_)_3_]·2H_2_O·6DMF (**2-ox**) (H_2_C_6_O_4_Cl_2_ = 2,5-dichloro-3,6-dihydroxo-1,4-benzoquinone) (Figure 7).^64^ Postsynthetic chemical reduction of the framework with cobaltocene in DMF led to (Cp_2_Co)_1.43_(Me_2_NH_2_)_1.57_[Fe_2_(C_6_O_4_Cl_2_)_3_]·(DMF)_4.9_ (**2-red**) via single crystal to single crystal transformation. The pristine MOF contains mixed-valence (C_6_O_4_Cl_2_)^2–/3–•^ in a 1:2 ratio while the reduced form has only the (C_6_O_4_Cl_2_)^3–•^ form. The room-temperature conductivity values are 1.4 × 10^–2^ S cm^–1^ and 5.1 × 10^–4^ S cm^–1^ for the oxidized and reduced forms of the MOF, respectively (Table 1). The loss of mixed valence states upon reduction is the main reason for the lower conductivity value observed in the reduced phase. The corresponding activation energies are 260 and 340 meV in **2-ox** and **2-red**, respectively.

Liu et al. have reduced the 2D MOF (Me_4_N)_2_[Mn_2_(Cl_2_dhbq)_3_] (**3-ox**) to Na_3_(Me_4_N)_2_[Mn_2_(C_6_O_4_Cl_2_^3–•^)_3_]·3.9THF (**3-red**) with sodium naphthalenide and then reoxidized the compound to Na(Me_4_N)[Mn_2_(C_6_O_4_Cl_2_)_3_]·5.5THF·0.8CH_3_CN (**3-reox**) with [Cp_2_Fe](BF_4_).^67^ Both oxidized products have a −2 oxidation state in the ligands, while the reduced form contains mixed-valence C_6_O_4_Cl_2_^3–^ ligands. The room-temperature electrical conductivity values are 1.14 × 10^–13^ and 1.45 × 10^–13^ S cm^–1^ for **3-ox** and **3-reox**, respectively, while **3-red** has a higher conductivity value of 2.27 × 10^–8^ S cm^–1^. The activation energy of the reduced MOF **3-red** (489 meV) is lower than that of the parent MOF **3-ox** (740 meV). We can conclude that, in this case, chemical reduction injects free electrons to the system, increasing the conductivity by 5 orders of magnitude.

Wentz et al. have synthesized a porous 3D MOF using the redox-active naphthalenediimide ligand. The reduced framework shows 10^6^ times higher conductivity than the original framework.^68^

Following the inner sphere redox-active metal–ligand pair, Clérac et al. have developed an interesting 2D layered metal–organic framework [Cr^III^Cl_2_(pyz)^•–^]_*n*_ (**4**) that shows both interesting ferrimagnetism and high electrical conductivity.^69^ It is formed by connecting metal centers with four pyrazine ligands while the chlorides occupy the *trans*-axial positions. The complex undergoes inner-sphere electron transfer to produce [Cr^III^Cl_2_(pyz)^•–^]_*n*_ (**4**) through electron transfer from metal to ligand to form Cr^3+^ and half-reduced (pyz_2_)^−^. This electronic configuration creates strong magnetic interaction between S = 3/2 Cr^III^ and delocalized S = 1/2 pyrazine spin, and [Cr^III^Cl_2_(pyz)^•–^]_*n*_ shows strong ferrimagnetic interaction below 55 K. The charge delocalization also provides a pathway for electron transport, and this compound shows a high electrical conductivity of 0.032 S cm^–1^ at room temperature (two-probe method using pressed pellet). Using the same strategy, they have prepared two other 2D layered materials: [V^II^Cl_2_(pyz)_2_]_*n*_ (**5**) and [Ti^II^Cl_2_(pyz)_2_]_*n*_ (**6**).^70^ Detailed analysis reveals that the former complex remains in its nonreduced state, while the latter undergoes inner-sphere electron transfer to produce [Ti^III^Cl_2_(pyz)^•–^]_*n*_ (**6**). Electrical conductivity measurement shows that the V-complex is insulating (σ = 10^–10^ S/cm) in nature while the Ti-complex shows metallic conductivity (σ = 5.3 S/cm) due to similar metal–ligand inner-sphere electron transfer. [Ti^III^Cl_2_(pyz)^•–^]*_n_* also shows a magnetoresistance of 25% at 1.8 K and ±9 T. Based on these results, they have established that electrical conductivity depends on the energy of the d-orbitals.

#### 2.1.2 Extended Conjugated Organic Ligands

**Scheme 1:**
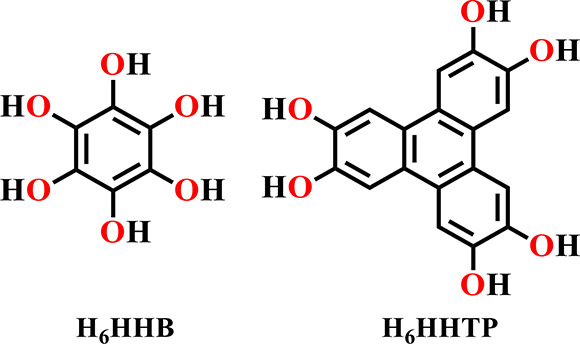
Hexahydroxybenzene (H_6_HHB) and Hexahydroxyterphenylene (H_6_HHTP) Ligands Used for Designing Conductive MOFs

**Figure 8:**
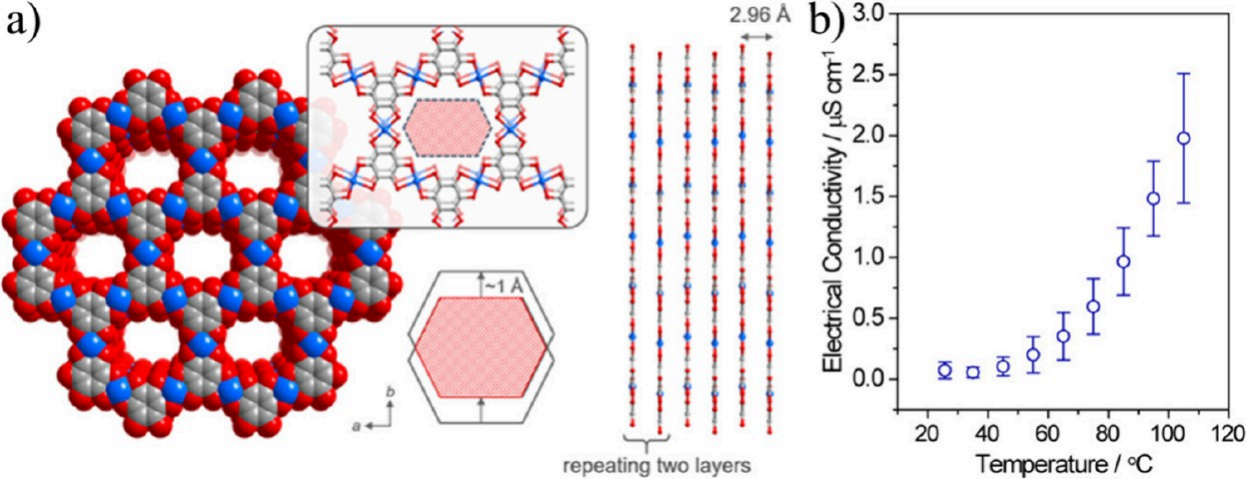
(a) Space-filling model of Cu_3_(HHB)_2_ (**7**) model. Color code: O = red, C = gray, and Cu = blue. (b) Electrical conductivity of Cu_3_(HHB)_2_ as a function of temperature. Reproduced from ref (71). Copyright 2018 American Chemical Society.

Another strategy to increase the electrical conductivity is the use of organic ligands having highly conjugated cores like triphenylene, phthalocyanine, napthalocyanine, etc. Thus, Park et al. synthesized MOF Cu_3_(HHB)_2_ (**7**) using the ligand hexahydroxybenzene (H_6_HHB, Scheme 1).^71^ This MOF shows a honeycomb layered structure with Cu^II^ ions in a four coordinated square planar geometry (Figure 8). Two-probe electrical conductivity measurements show that this MOF is a modest conductor with a room-temperature conductivity of 7.3 × 10^–8^ S cm^–1^, and the corresponding activation energy is 450 meV (Table 2). This conductivity value is a combination of both in-plane and out-of-plane conductivity of the MOF.

**Table 2:** Conducting MOFs Prepared with Extended Conjugated Ligands

#	Formula	Dim	σ (S cm^–1^) pressed pellet	σ (S cm^–1^) out-of-plane	σ (S cm^–1^) in-plane	*E*_a_ (meV)	ref.
**7**	Cu_3_(HHB)_2_	2D	7.3 × 10^–8^^a^	–	–	450	(71)
**8**	Cu_3_(HHTP)_2_	2D	0.02–0.2^b^	0.1^b^	–	–	(72−74)
**8-film**	Cu_3_(HHTP)_2_	2D	–	–	0.29^c^	130	(75)
**8-hex**	Cu_3_(HHTP)_2_	2D	–	1.5^d^	0.5^d^	–	(76)
**9**	Cu_3_(HHTP)(HHB)	2D	2.53 × 10^–5^^c^	–	–	–	(77)
**10**	Co_9_(HHTP)_4_	2D	0.1–0.003^e^	–	–	–	(78, 79)
**11**	Ni_9_(HHTP)_4_	2D	0.01–3 × 10^–6^^e^	–	–	–	(78, 79)

a Four-probe.

b Van der Pauw.

c Two-probe (thin film).

d Four- and two-probe on single crystals.

e Electrochemical.

A similar MOF, Cu_3_(HHTP)_2_ (**8**), with the extended conjugated ligand 2,3,6,7,10,11-hexahydroxytriphenylene (H_6_HHTP, Scheme 1) has been synthesized by Hmadeh and co-workers.^72^ The electrical conductivity measurements performed on single crystals of the MOF shows a very high conductivity of 0.1 S cm^–1^ at room temperature, similar to the values measured on powder and on a thin film of the MOF (in the range of 0.02 to 0.2 S cm^–1^).^73,74^ Song et al. have measured the electrical conductivity of thin films of Cu_3_(HHTP)_2_ (**8-film**) along the *ab*-plane with the four-probe method.^75^ These measurements show a high in-plane conductivity value of 0.29 S cm^–1^ with an activation energy of 130 meV (Table 2). Day and co-workers prepared hexagonal flakes of the 2D layers (**8-hex**) by a sonication-assisted liquid-phase exfoliation method.^76^ Four-probe conductivity measurement shows an out-of-plane conductivity of 1.5 S cm^–1^ while the in-plane conductivity is 0.5 S cm^–1^. Yao et al. synthesized a hybrid of Cu_3_(HHTP)_2_ and Cu_3_(HHB)_2_ with the formula Cu_3_(HHTP)(HHB) (**9**).^77^ Two-probe electrical conductivity measurements on pressed pellets show a moderate conductivity of 2.53 × 10^–5^ S cm^–1^, which is lower than the value of Cu_3_(HHTP)_2_ but much higher than that of Cu_3_(HHB)_2_. These results indicate that the presence of extended conjugated organic ligands facilitates the charge transport and results in highly conducting frameworks (Table 2).

Hmadeh et al. also reported two other MOFs with formula M_9_(HHTP)_4_, with M = Co (**10**) and Ni (**11**). In contrast to Cu_3_(HHTP)_2_, where the metal ions are four-coordinated square planar, in Co_9_(HHTP)_4_ and Ni_9_(HHTP)_4_, the metal ions present a hexacoordinated octahedral geometry with two extra coordination positions occupied by two water molecules. A second difference between the Cu and the Ni and Co derivatives is the ligand charge. Thus, in Co_9_(HHTP)_4_ and Ni_9_(HHTP)_4_, the average charge on the ligand is −4.5, while it is −3 in Cu_3_(HHTP)_2_. Electrical conductivity measurement shows that the conductivity values vary in the range of 0.1 to 0.003 S cm^–1^ and 0.01 to 3 × 10^–6^ S cm^–1^ for Co_9_(HHTP)_4_ and Ni_9_(HHTP)_4_, respectively.^74,78,79^

#### 2.1.3 Use of Hard/Soft Donor Atoms

Metal–donor atom overlap is one of the key factors for the *through-bond* and *through-layer* charge transport in MOFs. Well-matched energy levels, and consequent good overlap, between the metal and donor atoms can increase the charge delocalization.^42^ According to hard–soft acid–base (HSAB) principle, the hard–hard combination is rather ionic in nature while the soft–soft interaction leads to predominant covalency. Combination of hard atoms like O with soft transition metal atoms leads to poor metal–donor atom overlap due to the high energy difference of their orbitals. In contrast, the combination of soft transition metal ions at their lower oxidation states with ligands having soft donor atoms like P, S, Se, etc., can increase the metal–ligand overlap as well as the covalency. Thus, a proper metal–ligand combination can significantly enhance the electrical conductivity of MOFs.

##### O-Donor Ligands

**Figure 9:**
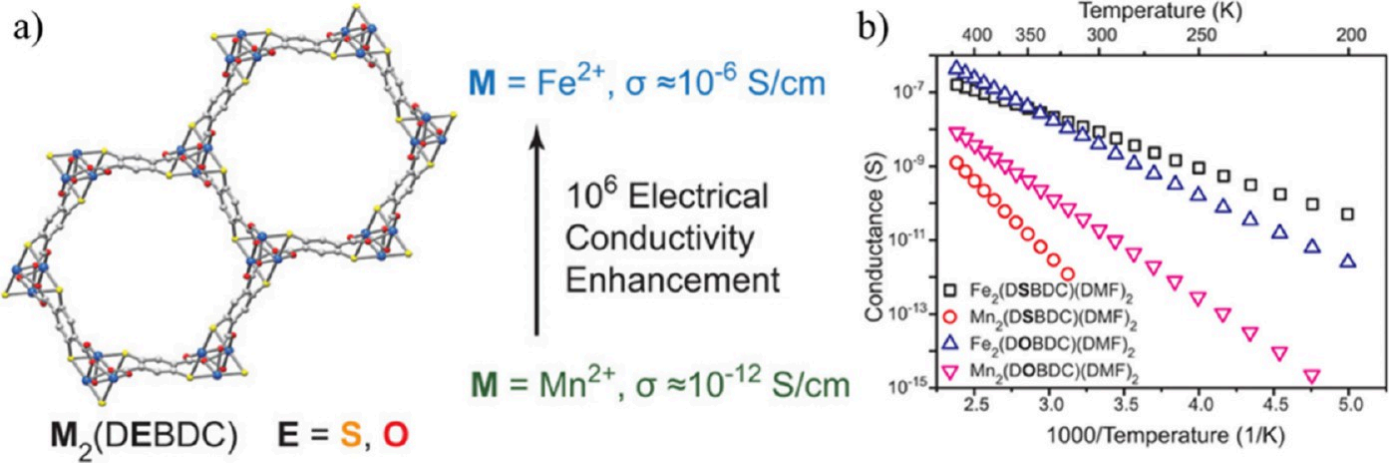
(a) Structure of M_2_(DEBDC) (E = S, O) MOFs. (b) Electrical conductivity as a function of 1/*T* for four different derivatives. Color code: C = gray, S = yellow, and M = blue. Reproduced from ref (80). Copyright 2018 American Chemical Society.

MOF-74 or CPO-27 are a special class of MOFs with the formula [M^II^_2_(DOBDC)] (where DOBDC = 2,5-dioxidobenzene-1,4-dicarboxylate and M^II^ = Mg, Mn, Fe, Co, Ni, and Zn) that contains (−M–O−)_∞_ chains as the secondary building unit (Figure 9).^80,81^ These MOFs show through-bond charge transport along the infinite M–O bonds. Isomorphous MOFs with (−M–S−)_∞_ chains are obtained by using thiol-decorated H_4_DSBDC (2,5-disulfhydrylbenzene-1,4-dicarboxylic acid). Sun et al. have reported that the conductivity of the [M^II^_2_(DOBDC)] MOFs (M^II^ = Mg, Mn, Co, Ni, and Zn) vary in the range of 1.4 × 10^–14^ to 3 × 10^–13^ S cm^–1^, whereas the thiolated MOF [Mn_2_(DSBDC)] (**12**) shows a 10 times higher conductivity (2.5 × 10^–12^ S cm^–1^) than [Mn_2_(DOBDC)] (**13**) (3.9 × 10^–13^ S cm^–1^). In the Fe^II^ derivative, the conductivity of [Fe_2_(DSBDC)] (**14**) (3.9 × 10^–6^ S cm^–1^) is also 10 times higher than that of the phenolate analogue [Fe_2_(DOBDC)] (**15**) (3.2 × 10^–7^ S cm^–1^) (Table 3). This result indicates that an efficient orbital overlap between the metal d-orbitals and ligand p-orbitals by matching their energy levels is an appropriate strategy to promote electrical conductivity in MOFs. Thus, the (−M–S−)_*n*_ chains facilitate the electron transport better than the (−M–O−)_*n*_ chains. The higher conductivity shown by the Fe-based MOFs compared to the Mn ones is attributed to the presence of loosely bound β-spin d-electrons in Fe^II^, not present in Mn^II^.

**Table 3:** Conducting MOFs with O/S-Donor Atom-Based Organic Ligands

#	MOF	Ligand	Dim	σ (S cm^–1^)	ref
**12**	[Mn_2_(DSBDC)]	DSBDC	3D	3.9 × 10^–13^^a^	(80, 81)
**13**	[Mn_2_(DOBDC)]	DOBDC	3D	2.5 × 10^–12^^a^	(80)
**14**	[Fe_2_(DSBDC)]	DSBDC	3D	3.9 × 10^–6^^a^	(80)
**15**	[Fe_2_(DOBDC)]	DOBDC	3D	3.2 × 10^–7^^a^	(80)

a Two-probe on pressed pellets

##### N-Donor Ligands

**Scheme 2:**
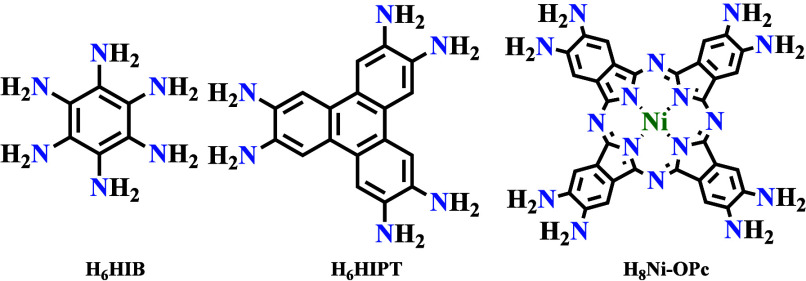
N-Donor-Based Organic Ligands for Designing Conducting MOFs

Amine-functionalized conjugated organic ligands such as hexaiminobenzene (HIB, Scheme 2),^82^ hexaiminotriphenylene (H_6_HITP, Scheme 2), and octaiminophthalocyanine (H_8_OIPc, Scheme 2) have also been used to prepare conducting MOFs.

**Figure 10:**
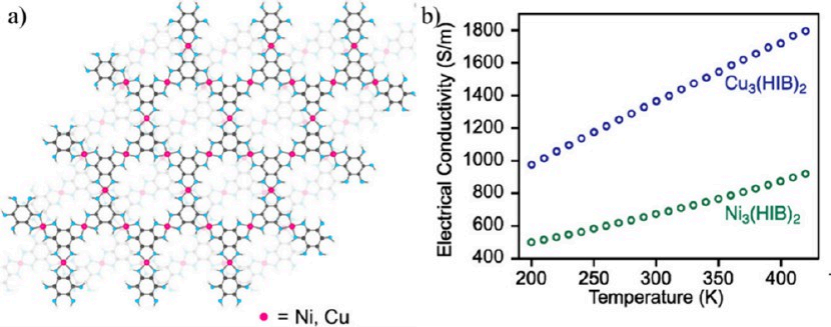
(a) Calculated structures of M_3_(HIB)_2_ (M = Ni and Cu) (b) Variable-temperature electrical conductivity of pressed pellets of M_3_(HIB)_2_ measured by the van der Pauw method. Color code: C = gray; M = pink, and N = blue. Reproduced from ref (83). Copyright 2017 American Chemical Society.

Thus, Dou et al. have reported metallic conductivity in Ni_3_(HIB)_2_ (**16**) and Cu_3_(HIB)_2_ (**17**). In both cases, hexagonal honeycomb layers are stacked in a slipped-parallel fashion (Figure 10).^83^ Electrical conductivity measurements on pressed pellets by the van der Pauw method show conductivity values in the range of 0.7 to 10 S cm^–1^ and 0.11 to 0.7 S cm^–1^ for Ni_3_(HIB)_2_ (**16**) and Cu_3_(HIB)_2_ (**17**), respectively. Park et al. reported conductivity values in the range of 0.1 to 1.57 S cm^–1^ for Co_3_(HIB)_2_ (**18**), measured by the two-probe method on pressed pellets.^84^ Lee and co-workers reported very high conductivity values for the Fe_3_(HIB)_2_ (**19**) and Mn_3_(HIB)_2_ (**20**) derivatives of this series (Table 4).^85^

**Table 4:** Conducting MOFs with N-Donor Atom-Based Organic Ligands

#	Complex	Ligand	Dim	σ (S cm^–1^)	ref.
**16**	Ni_3_(HIB)_2_	HIB	2D	0.7–10^a^	(83)
**17**	Cu_3_(HIB)_2_	HIB	2D	0.11–0.7^a^	(83)
**18**	Co_3_(HIB)_2_	HIB	2D	0.1–1.57^b^	(84)
**19**	Fe_3_(HIB)_2_	HIB	2D	150^a^	(85)
**20**	Mn_3_(HIB)_2_	HIB	2D	108^a^	(85)
**21**	Ni_3_(HITP)_2_	HITP	2D	10^c^	(86)
**21-film**	Ni_3_(HITP)_2_	HITP	2D	40^c^	(86)
**21**	Ni_3_(HITP)_2_	HITP	2D	150^d^	(76)
**22**	Co_3_(HITP)_2_	HITP	2D	8 × 10^–4^^e^	(89)
**23**	Co_*x*_Ni_3–*x*_(HITP)_2_	HITP	2D	3.2 × 10^–2^^e^	(89)
**24**	[Ni_2_(Ni-OIPc)]	Ni-OIPC	2D	0.2^f^	(90)

a Van der Pauw on pressed pellet.

b Four-probe on pressed pellet.

c Impedance on pressed pellet.

d Two- and four-probe on single crystal.

e Four-probe on pressed pellet.

f Four-probe on thin film.

The ligand hexaiminotriphenylene (HITP, Scheme 2) has also been used to prepare several conducting MOFs. Sheberla et al. reported metallic-like conductivity in the MOF Ni_3_(HITP)_2_ (**21**),^86^ where the honeycomb hexagonal layers show a near-eclipsed packing. Four-probe electrical conductivity measurements on thin films (**21-film**) and pressed pellets (**21**) of this MOF show room-temperature conductivities of 40 and 10 S cm^–1^, respectively, and a semiconducting behavior, although the charge transport phenomena is not clear. Day et al. have measured electrical conductivity on single crystals of Ni_3_(HITP)_2_ and found a value of ∼150 S cm^–1^ at 295 K and nonzero conductivity at 0.3 K.^74^ Charge carrier mobility measurements of thin films of the Ni_3_(HITP)_2_ MOF show a value of 40 cm^2^ V^–1^ s^–1^.^87^ The high conductivity arises from the contribution of both in-plane and out-of-plane charge transport. Campbell et al. have found a conductivity value of 0.2 S cm^–1^ on pressed pellets of Ni_3_(HITP)_2_ measured by the two-probe method.^88^ Lian and co-workers have synthesized the Co_3_(HITP)_2_ (**22**) and Co_*x*_Ni_3–*x*_(HITP)_2_ (**23**) derivatives. The electrical conductivity of Co_3_(HITP)_2_ (**22**) is very small (8 × 10^–4^ S cm^–1^), whereas the mixed-metal MOF shows a much higher conductivity (0.032 S cm^–1^) that increases with increasing the Ni content.^89^

Finally, Jia and co-workers have synthesized the highly conjugated 2D MOF [Ni_2_(Ni-OIPc)] (**24**) using the metallophthalocyanine-based organic linker Ni-OIPC (Scheme 2).^90^ Four-probe electrical conductivity measurement on a pressed pellet of the sample shows a high room-temperature conductivity value of ∼0.2 S cm^–1^ (Table 4).

##### S-Based Ligands

**Scheme 3:**
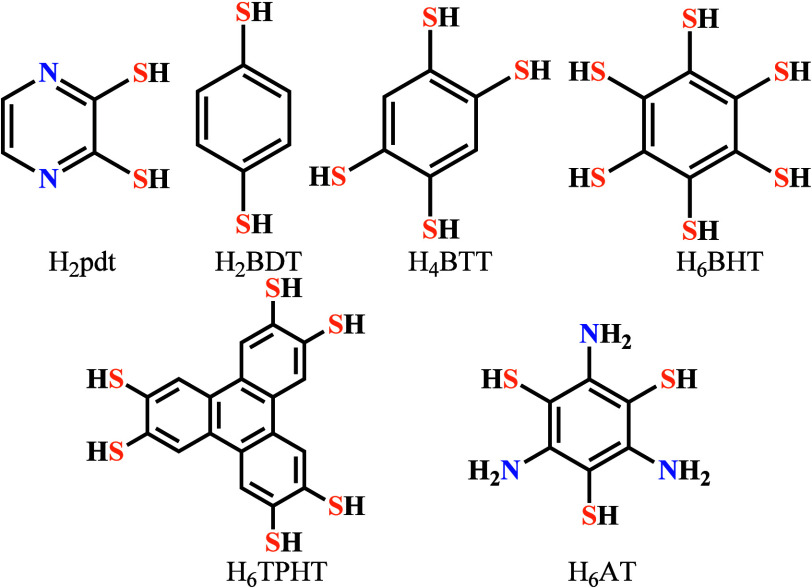
Thiol- and Mixed N-, S-Donor-Based Ligands for Designing Conductive MOFs

Different thiol-based ligands have gained significant attention in designing conductive MOFs (Scheme 3). Takaishi et al. used 2,3-pyrazinedithiolate (pdt) to construct a porous 3D MOF formulated as Cu[Cu(pdt)_2_] (**25**) that shows a conductivity value of 6 × 10^–4^ S cm^–1^ at 300 K.^38^ The temperature dependence of the electrical conductivity indicates that this MOF is a semiconductor with an activation energy of 193 meV.

Turner et al.^91^ synthesized three different MOFs using three different ditopic, tetratopic, and hexatopic thiol-based ligands. Thus, with 1,4-benzenedithiol they prepared the 3D MOF [Pb_3_(SC_6_H_4_S)_3_(en)_2_]_*n*_ (**26**), where the metal centers are connected by SC_6_H_4_S units. They also prepared a second 3D MOF, [Pb_2_(S_2_C_6_H_2_S_2_)(en)]_*n*_ (**27**), with 1,2,4,5-benzenetetrathiol. Electrical conductivity measurements on pressed pellets of these 3D MOFs show insulating behavior with conductivity values below 10^–12^ S cm^–1^. Finally, they also prepared a 2D MOF formulated as [Pb_3_(BHT)]_*n*_ (**28**) with the ligand benzenehexathiol (H_6_BHT, Scheme 3). This MOF shows an electrical conductivity of 10^–6^ S cm^–1^ at room temperature and an activation energy of 370 meV.

Chen and co-workers synthesized two different 2D MOFs formulated as Ag_3_BHT_2_ (**29**) and Au_3_BHT_2_ (**30**) using benzenehexathiol (H_6_BHT, Scheme 3) as the ligand with a liquid–liquid interfacial method.^92^ The honeycomb hexagonal layers are almost eclipsed for Ag_3_BHT_2_ (**29**) but slightly displaced for Au_3_BHT_2_ (**30**). The electrical conductivity of the Ag-based MOF is higher than the Au one, and the conductivity is dependent on the thickness of the films. Thus, for thin (≈ 0.276 μm) and thick (≈ 64 μm) films, the electrical conductivity values change from 363 to 19.8 S cm^–1^, respectively, in the Ag_3_BHT_2_ MOF. For the Au_3_BHT_2_ MOF, these values are 1.12 × 10^–4^ (for thin films of ≈0.325 μm) and 8.07 × 10^–5^ S cm^–1^ (for thick films of ≈89 μm). The large differences in the conductivity values can be easily explained by the better packing of the layers in the Ag-MOF (almost eclipsed), compared to the Au-MOF (slightly alternated), which leads to stronger interlayer π–π interactions in the Ag-MOF, responsible for the much higher electrical conductivity of the Ag-MOF.

**Figure 11:**
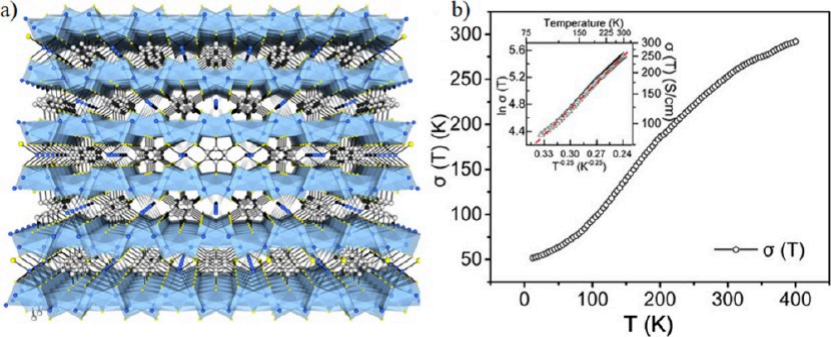
(a) View of the structure of [Ag_5_(BHT)]_*n*_ (**31**). Color code: Ag = blue, S = yellow, and C = gray. The Ag-centered tetrahedra are shown in pale blue. (b) Electrical conductivity of a thin film of [Ag_5_(BHT)]_*n*_ (**31**) as a function of temperature. Reproduced from ref (93). Copyright 2017 American Chemical Society.

Using the liquid–liquid interface method, Huang et al. have synthesized another BHT-based 2D MOF (Figure 11) with Ag, with the molecular formula [Ag_5_(BHT)]_*n*_ (**31**).^93^ Four-probe electrical conductivity measurements of thin films of this MOF show very high conductivity values of ≈250 S cm^–1^ at room temperature and an increase of the conductivity with temperature, typical of semiconductors. The activation energy of the MOF also increases with temperature. This high conductivity is based on both Ag–S bonding in combination with the hopping process between neighboring Ag-BHT nanocrystals within the thin film. The same group also prepared a Cu-MOF with the same ligand, formulated as [Cu_3_(BHT)]_*n*_ (**32**).^58^ This MOF presents the highest conductivity value reported to date in a MOF: 750–1580 S cm^–1^, measured on highly crystalline thin films prepared by a liquid–liquid interface method. Four-probe conductivity measurements on thin films of this MOF with different thickness show that the conductivity values do not vary with the thickness of the films. The conductivity of these films decreases when the temperature is lowered and reaches a minimum value of 1360 S cm^–1^ at 2 K. The activation energy rises continuously with temperature, from 0.12 meV at 40 K to 2.06 meV at 300 K.^58^ Furthermore, this 2D MOF shows the appearance of superconductivity at 0.25 K at ambient pressure.^59^

**Figure 12:**
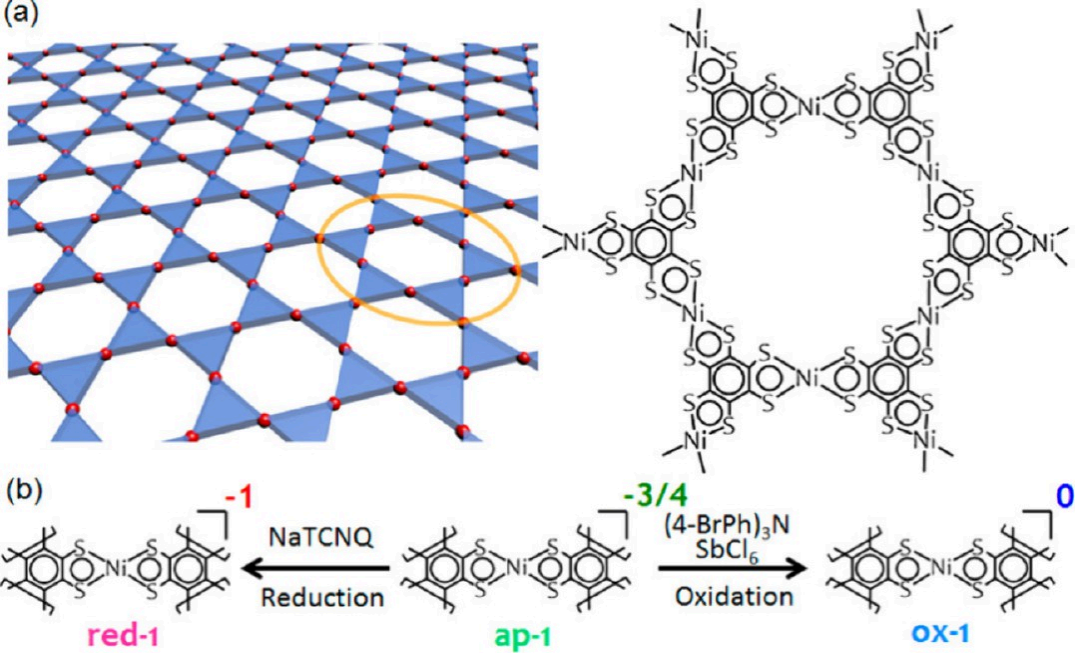
(a) Illustration of the chemical structure of the Ni_3_(BHT)_2_ (**33**) nanosheets. (b) Schematic illustration of the redox control in **33**. Reproduced from ref (53). Copyright 2014 American Chemical Society.

Kambe et al. have established that the electrical conductivity of the 2D metal-dithiolene MOF Ni_3_(BHT)_2_ (**33**) varies with the oxidation state.^39,53^ For the pristine MOF Ni_3_(BHT)_2_ (**33**), both the IR and XPS analysis reveal that the average charge on the metal center is −3/4. These authors, therefore, attempted to oxidize and reduce the framework to get pure 0 and −1 oxidation states (Figure 12). IR and XPS studies show that the reduced framework obtained using NaTCNQ shows a charge of −1 (**33-red**).^39^ Two-probe electrical conductivity measurements on pressed pellets of the parent and reduced MOFs show conductivity values of 0.15 and 6.7 × 10^–3^ S cm^–1^ at 298 K. The oxidation of the parent MOF using tris(4-bromophenyl)ammonium hexachloroantimonate leads to a sample with a much higher conductivity of 160 S cm^–1^, measured by the four-probe van der Pauw method to avoid grain boundary effect and contact resistance.^53^ In fact, with the four-probe method, the conductivity of the parent MOF increases from 0.15 to 2.8 S cm^–1^.

Pal et al. have prepared another similar MOF: [Pd_3_(BHT)_2_] (**34**) by a liquid–liquid interface method, although it was contaminated with PdNPs.^94^ Accordingly, they used K_3_[Fe(CN)_6_] as an oxidizing agent to prevent the formation of PdNPs. Four-probe electrical conductivity measurements on pressed pellets of the sample shows a conductivity at room temperature of 2.80 × 10^–2^ S cm^–1^.

Another highly extended conjugated thiol-based organic ligand that has been used to prepare conducting MOFs is triphenylenehexathiol (H_6_TPHT, Scheme 3). Cui et al. reported that the reaction between H_6_TPHT with Pt(CH_3_CN)_2_Cl_2_ produces a red-colored compound under N_2_ atmosphere that turned black upon exposure to open air.^95^ ICP analysis of the compound reveals that the formula of the compound is (Pt)_1.5_(Na)_0.9_TPHT (**35**) and the 2D framework is anionic in nature. The anionic framework is easily oxidized by I_2_ to form Pt_3_(TPHT)_2_ (**36**). The hexagonal honeycomb layers are stacked in a staggered conformation. Two-probe electrical conductivity measurement shows conductivity values of 2.47 × 10^–6^ and 1.09 × 10^–6^ S cm^–1^ for TPHT(Pt)_1.5_(Na)_0.9_ (**35**) and Pt_3_(TPHT)_2_ (**36**), respectively.

**Figure 13:**
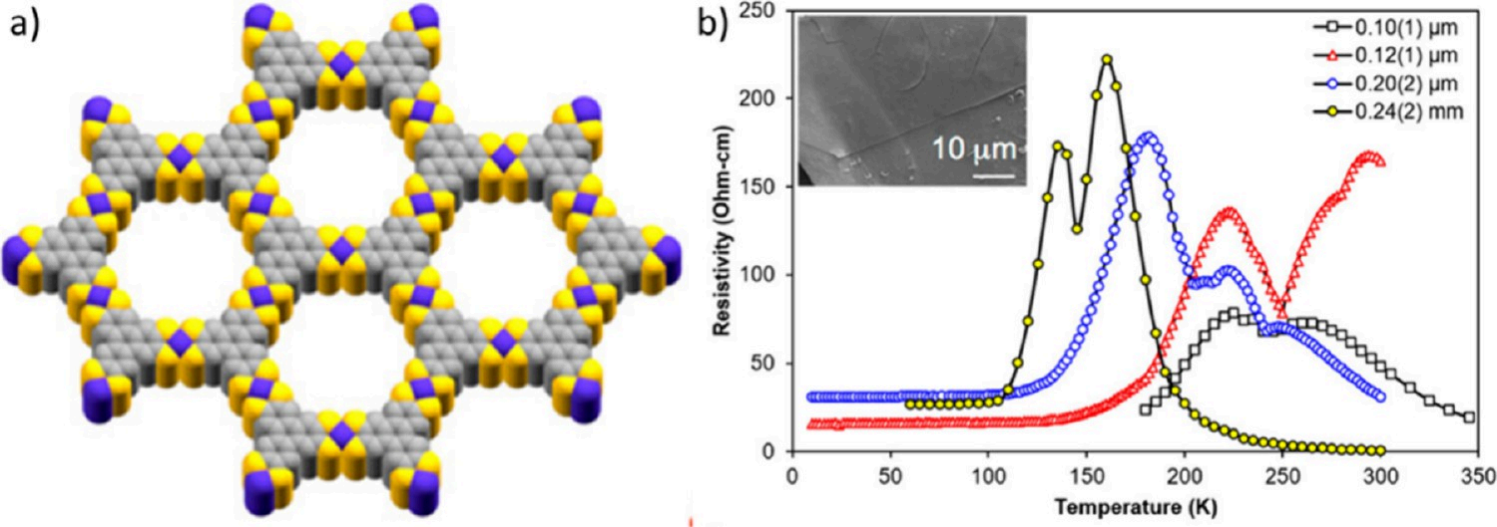
(a) 2D layered structure of the MOF **37**. (b) Variable-temperature resistivity data for the solid MOF pressed in a pellet of 0.24(2) mm thickness (yellow, scaled down 10^5^×) and films of the MOF with thicknesses of 0.10(1) (black), 0.12(1) (red), and 0.20(2) (blue) μm deposited on glass supports. Inset in panel (b): a SEM image of one film. Color code: C = gray, S = yellow, and Co = blue. Reproduced from ref (96). Copyright 2014 American Chemical Society.

Clough et al. synthesized another similar 2D MOF formulated as Co_3_(TPHT)_2_ (**37**) where the hexagonal honeycomb layers are eclipsed (Figure 13).^96^ Four-probe van der Pauw measurements show that the electrical conductivity of a pressed pellet of the MOF with thickness 0.24(2) mm is 1.4 × 10^–3^ S cm^–1^ with an activation energy of 173 meV. Interestingly, this MOF shows a transition from a semiconductor to metallic state on lowering the temperature from 130 to 50 K (Figure 13), although this transition is dependent on the thickness of the pressed pellets and might be an artifact. Such high electrical conductivity is due to both strong metal–ligand orbital overlap and highly conjugated organic ligand present within the 2D layered material.

Dong and co-workers have synthesized another 2D MOF formulated as Fe_3_(TPHT)_2_(NH_4_)_3_ (**38**) by liquid–liquid interfacial method with time-dependent variable thicknesses.^97^ In this compound, the honeycomb hexagonal layers show a tilt between them. Exposure to open air leads to oxidation of both metal and TPHT ligand, which consequently converts this material from semiconducting to metallic conductor. Four-point van der Pauw conductivity measurements show a conductivity value of 3.4 × 10^–2^ S cm^–1^ at 300 K (Table 5).

**Table 5:** Conducting MOFs with S, Se, and Mixed Donor-Atom-Based Organic Ligands

#	Complex	Ligand	Dim	σ (S cm^–1^)	*E*_a_ (meV)	ref
**25**	Cu[Cu(pdt)_2_]	pdt	3D	6 × 10^–4^^a^	193	(38)
**26**	[Pb_3_(SC_6_H_4_S)_3_(en)_2_]_*n*_	BDT	3D	<10^–12^^b^		(91)
**27**	[Pb_2_(S_2_C_6_H_2_S_2_)(en)]_*n*_	BTT	3D	<10^–12^^b^		(91)
**28**	[Pb_3_(BHT)]_*n*_	BHT	3D	10^–6^^b^	370	(91)
**29**	Ag_3_BHT_2_	BHT	2D	363^c^		(92)
**29**	Ag_3_BHT_2_	BHT	2D	19.8^c^		(92)
**30**	Au_3_BHT_2_	BHT	2D	1.12 × 10^–4^^c^		(92)
**30**	Au_3_BHT_2_	BHT	2D	8.07 × 10^–5^^c^		(92)
**31**	[Ag_5_(BHT)]_*n*_	BHT	3D	250^c^		(93)
**32**	[Cu_3_(BHT)]_*n*_	BHT	2D	750–1580^c^	2.06/120^h^	(58)
**33**	[Ni_3_(BHT)_2_] (charge –3/4)	BHT	2D	0.15^d^		(39)
**33-red**	[Ni_3_(BHT)_2_] (charge −1)	BHT	2D	6.7 × 10^–3^^d^		(39)
**33**	[Ni_3_(BHT)_2_] (charge –3/4)	BHT	2D	2.8^e^		(53)
**33-red**	[Ni_3_(BHT)_2_] (charge −1)	BHT	2D	160^e^		(53)
**34**	[Pd_3_(BHT)_2_]	BHT	2D	2.80 × 10^–2^^c^		(94)
**35**	(Pt)_1.5_(Na)_0.9_TPHT	TPHT	2D	2.47 × 10^–6^^b^		(95)
**36**	Pt_3_(TPHT)_2_	TPHT	2D	1.09 × 10^–6^^b^		(95)
**37**	Co_3_(TPHT)_2_	TPHT	2D	1.4 × 10^–3^^e^	173	(96)
**38**	Fe_3_(TPHT)_2_(NH_4_)_3_	TPHT	2D	3.4 × 10^–2^^c^		(97)
**39**	K_3_Fe_2_[PcFe-O_8_]	PcFe-O_8_	2D	0.2^f^		(98)
**40**	[Ni(AT)_2_]	AT^3–^	2D	3 × 10^–6^^e^		(100)
**41**	[Ni(IT)_2_]	IT^6–^	2D	0.1^g^		(99)
**42**	[Cu_3_(C_6_Se_6_)]_*n*_	BHS	2D	110^f^		(101)
**43**	PhSeAg	PhSe	2D	2.93 × 10^–11^^b^		(102)

a Two-probe on single crystal.

b Two-probe on pressed pellet.

c Four-probe on thin film.

d Two-probe on thin film.

e Van der Pauw on thin film.

f Four-probe on pressed pellet.

g Van der Pauw on pressed pellet.

h *E*_a_ at 40 K.

Yang et al. used a highly conjugated thiol-based organic ligand containing a coronene core to prepare a conducting MOF formulated as K_3_Fe_2_[PcFe-O_8_] (**39**), with PcFe-O_8_ = (2,3,9,10,16,17,23,24-octahydroxyphthalocyaninato)iron.^98^ This MOF shows alternating planar metal–organic layers. The conductivity of the sample, measured with the van der Pauw method on pressed pellets, shows a conductivity value of 0.2 S cm^–1^ at room temperature (Table 5).

Sun et al. used a mixed donor ligand 1,3,5-triaminobenzene-2,4,6-trithiol (H_6_AT, Scheme 3) having alternate thiol and amine groups to synthesize two different conducting MOFs, [Ni(AT)_2_] (**40**) and [Ni(IT)_2_] (**41**) (IT = 1,3,5-triiminobenzene-2,4,6-trithiolate), by varying the reaction conditions.^99,100^ The electrical conductivity of these MOFs, measured by the van der Pauw method, are 0.1 S cm^–1^ and 3 × 10^–6^ S cm^–1^ for [Ni(IT)_2_] (**41**) and [Ni(AT)_2_] (**40**), respectively.

**Figure 14:**
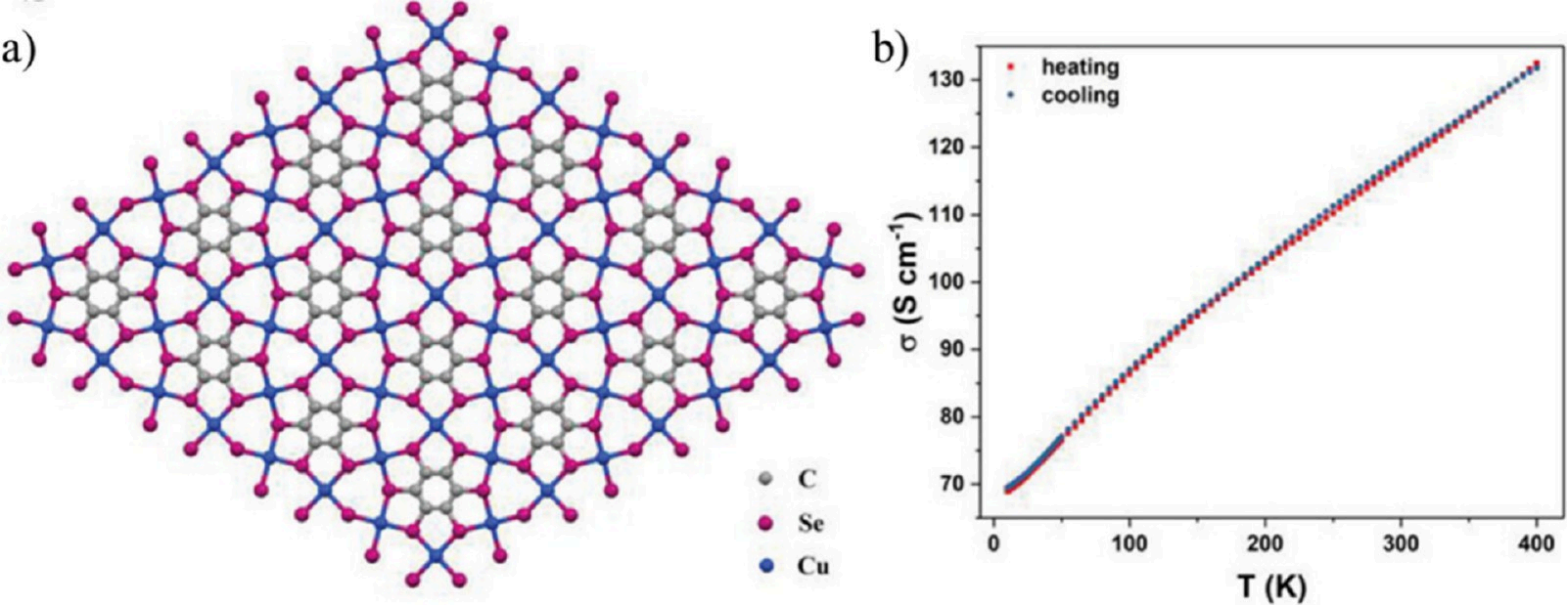
(a) 2D lattice of [Cu_3_(C_6_Se_6_)]_*n*_ (**42**). (b) Temperature dependence of the electrical conductivity of a [Cu_3_(C_6_Se_6_)]_*n*_ pellet. Color code: C = gray, Cu = blue, and O = red. Reproduced with permission from ref (101). Copyright 2019 John Wiley and Sons.

Although less common, Se-based ligands have also been used to design conducting MOFs. Thus, Cui et al. have synthesized a 2D MOF formulated as [Cu_3_(C_6_Se_6_)]_*n*_ (**42**) that presents honeycomb hexagonal layers with an eclipsed stacking (Figure 14).^101^ Four-probe electrical conductivity measurements on pressed pellets of the sample show a very high conductivity of ∼110 S cm^–1^ at 300 K that increases with temperature from 10 to 400 K, showing a semiconducting behavior. Enriched metal–ligand overlap between copper atoms and the Se atoms of the ligand provides the efficient charge transport pathway through the 2D metal–organic layer.

Another example of Se-based conducting MOF was prepared by Huang et al., which synthesized a special type of MOF formulated as PhSeAg (**43**), containing inorganic layers of AgSe with phenyl groups on both sides of the inorganic layer. Two-probe conductivity measurement reveals a conductivity at room temperature of 2.93 × 10^–11^ that increases to 3.95 × 10^–8^ S cm^–1^ at 418 K, indicative of a semiconducting behavior.^102^

#### 2.1.4 Mixed-Valence Metal Ions

As already described for the mixed-valence ligands, metal ions with tunable valences are also utilized to design conducting MOFs (Table 6) since the redox behavior of metal cations inside the MOFs may provide a pathway for electron transfer. One example is the MOF Cu[Cu(pdt)_2_] (**25**) (pdt = 2,3-pyrazinedithiolate) reported by Takaishi et al. in 2009. This mixed-valence MOF contains Cu^I^ cations and [Cu^III^(pdt)_2_]^−^ anions and shows a conductivity of 6 × 10^–4^ S cm^–1^ at 300 K.^38^ Okubo et al. reported a semiconductor based on a mixed-valence Cu^I^–Cu^II^ coordination polymer formulated as [Cu_3_^I^Cu^II^Br_3_(3,5-Dmpip-dtc)_2_]_*n*_ (**44**) (3,5-Dmpip-dtc = 3,5-dimethylpiperidine dithiocarbamate). This MOF has a room-temperature conductivity of 6.5 × 10^–8^ with an activation energy of 390 meV.^103^ In 2016, Wu et al. reported the mixed-valence semiconducting 3D MOF [Cu^Ι^Cu_2_^ΙΙ^(DCTP)_2_](NO_3_)·1.5DMF (**45**) (where DCTP = 4′-(3,5-dicarboxyphenyl)-4,2′:6′,4″-terpyridine) with a narrow bandgap and photocatalytic hydrogen evolution and degradation of organic dyes, based on photogenerated electrons and holes.^104^

**Table 6:** Conducting MOFs with Mixed-Valence Metal Ions

#	Complex	Redox couple	Dim	σ (S cm^–1^)	*E*_a_ (meV)	ref
**25**	Cu[Cu(pdt)_2_]	Cu^I^/Cu^III^	3D	6 × 10^–4^^a^		(38)
**44**	[Cu_3_^I^Cu^II^Br_3_(3,5-Dmpip-dtc)_2_]_n_	Cu^I^/Cu^II^	2D	6.5 × 10^–8^^b^	380	(103)
**45**	[Cu^Ι^Cu_2_^ΙΙ^(DCTP)_2_](NO_3_)·1.5DMF	Cu^I^/Cu^II^	3D	–	1050	(104)
**46**	Fe_2_(BDT)_3_	Fe^II^/Fe^III^	3D	6 × 10^–5^^a^		(105)
**46**	Fe_2_(BDT)_3_ after 7 days	Fe^II^/Fe^III^	3D	0.3^a^		(105)
**46**	Fe_2_(BDT)_3_ after 30 days	Fe^II^/Fe^III^	3D	1.2^a^	160	(105)
**47**	[Fe(tri)_2_(BF_4_)_0.33_]	Fe^II^/Fe^III^	3D	0.3^c^		(107)
**48**	[(H_3_O)(H_2_O)(phenazine)_3_] [Fe^II^Fe^III^(C_6_O_4_Cl_2_)_3_]·12H_2_O	Fe^II^/Fe^III^	2D	0.03;^a^^,^^d^ 1 × 10^–4^^a^^,^^e^	≈ 118	(55)
**49**	[(H_3_O)(H_2_O)(phenazine)_3_] [Fe^II^Fe^III^(C_6_O_4_Br_2_)_3_]·12H_2_O	Fe^II^/Fe^III^	2D	0.003;^a^^,^^d^ 1 × 10^–5^^a^^,^^e^	≈ 108	(55)

a Two-probe on single crystal.

b Impedance on pressed pellet.

c Two-probe on pressed pellet.

d Parallel to the layers.

e Perpendicular to the layers.

Sun and co-workers have attempted to partially oxidize iron (from Fe^2+^ to Fe^3+^) on single crystals of MOF Fe_2_(BDT)_3_ (**46**), where H_2_BDT = 5,5′-(1,4-phenylene)bis(1H-tetrazole), upon exposure to ambient atmosphere.^105^ The electrical conductivity of the as-synthesized red crystals is 6 × 10^–5^ S cm^–1^ at 296 K, but the conductivity value increases with time when exposed to air. After 7 days, the conductivity values increase to 0.3 S cm^–1^ and after 30 days to 1.2 S cm^–1^ (Table 6). The temperature dependence of the conductivity shows a classical Arrhenius-type semiconducting behavior with an activation energy of 160 meV. This MOF shows how the presence of a mixed-valence state reduces the activation barrier for charge transfer and increases the electrical conductivity up to 5 orders of magnitude,^106^ confirming that the use of redox-active metals within a MOF has a similar effect to the use of redox-active ligands.

**Figure 15:**
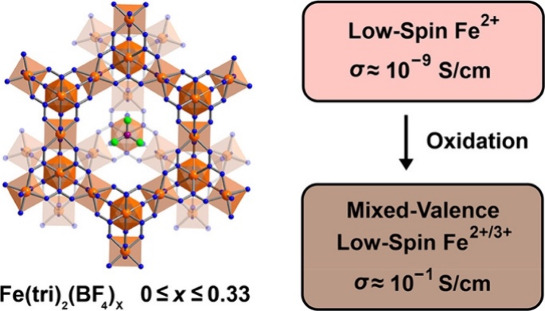
Modulation of the electrical conductivity of an Fe-containing MOF due to change in the oxidation states. Color code: C = gray, Fe = orange, N = blue, and Cl = green. Reproduced from ref (107). Copyright 2018 American Chemical Society.

Park et al. also reported the gradual increase in the electrical conductivity of the Fe^II^-containing MOF [Fe(tri)_2_] (tri = 1,2,3-triazolate) by chemically oxidizing it to the mixed-valence MOF [Fe(tri)_2_(BF_4_)_*x*_], with *x* = 0.09, 0.22, and 0.33 (**47**).^107^ The experimental results show that the derivative [Fe(tri)_2_(BF_4_)_0.33_] (**47**) has a high conductivity (0.3 S cm^–1^) and the electron transfer between Fe^II^/^III^ ions is the main reason behind such phenomenon (Figure 15).

Gómez-García et al. synthesized two different mixed-valence MOFs formulated as [(H_3_O)(H_2_O)(phenazine)_3_][Fe^II^Fe^III^(C_6_O_4_X_2_)_3_]·12H_2_O with X = Cl (**48**) and Br (**49**), having two different halide-substituted anilato ligands. These layered MOFs contain mixed-valence Fe^II^/Fe^III^ ions as shown by EPR and structural data analysis and, besides a magnetic ordering at low temperatures, show high in-plane electrical conductivities of 0.03 and 0.003 S cm^–1^, respectively.^55^

#### 2.1.5 Electronic Structure and Size of Metal Ions

There are some MOFs where the electrical conductivity (and even the conduction mechanism) can be modulated by changing the metal cations since their electronic structure and size may modulate the intermolecular contacts and, accordingly, the electronic conductivity. Long et al. have recently shown a very interesting case of three isostructural MOFs where the electrical conductivity mechanism depends on the electronic structure of the metal ion (Table 7). Thus, they have prepared three isostructural 2D anilato-based frameworks (H_2_NMe_2_)_2_[M_2_(C_6_O_4_Cl_2_)_3_] with M = Ti (**50**) and V (**51**) and (H_2_NMe_2_)_1.5_[Cr_2_(C_6_O_4_Cl_2_)_3_] (**52**).^108^ The room-temperature electrical conductivities of these Cr, Ti, and V-containing MOFs, measured under Ar atmosphere with the two-probe technique, are 1.2 × 10^–4^, 2.7 × 10^–3^, and 0.45 S cm^–1^ respectively. The thermal dependence of the conductivity with temperature show that the conductivity of the Ti and Cr-MOFs is due to redox-hopping between neighboring ligands in different valence states, rather than through excitation into a delocalized band. The activation energies of the Ti and Cr-based MOFs were calculated, using the Arrhenius model, as 270 and 440 meV, respectively. In contrast, the V-containing MOF follows a variable-range hopping (VRH) mechanism for charge transport with activation energies of 64 meV at 300 K and 11 meV at 20 K. The variable-temperature conductivity of the V-MOF was satisfactorily fit with the Efros–Shklovskii variable-range hopping model: 

**Table 7:** Conducting MOFs with Cation Size and Electronic Structure Dependent Conductivity

#	Complex	r (M^*n*+^) (pm)	Dim	σ (S cm^–1^)	*E*_a_ (meV)	ref
**50**	(H_2_NMe_2_)_2_[Ti_2_(C_6_O_4_Cl_2_)_3_]		3D	1 × 10^–4^^a^	270	(108)
**51**	(H_2_NMe_2_)_2_[Cr_2_(C_6_O_4_Cl_2_)_3_]		3D	2.7 × 10^–3^^a^	440	(108)
**52**	(H_2_NMe_2_)_2_[V_2_(C_6_O_4_Cl_2_)_3_]		3D	0.45^a^	64 (300 K); 11 (20 K)	(108)
**53**	Mn_2_(TTFTB)	88	3D	3.95 × 10^–6^^b^		(109)
**54**	Co_2_(TTFTB)	88.5	3D	1.49 × 10^–5^^b^		(109)
**55**	Zn_2_(TTFTB)	97	3D	8.64 × 10^–5^^b^		(109)
**56**	Cd_2_(TTFTB)	107	3D	2.86 × 10^–4^^b^		(109)

a Two-probe on pressed pellet.

b Two-probe on single crystal.

Another interesting example of cation-dependent conductivity in MOFs was reported by Park et al. in a series of four isostructural MOFs formulated as M_2_(TTFTB) (TTFTB = tetrathiafulvalene-tetrabenzoate) with M^2+^ = Mn^2+^ (**53**), Co^2+^ (**54**), Zn^2+^ (**55**), and Cd^2+^ (**56**). These authors observed that the electrical conductivity depends on the cation size (Table 7).^109^ Two-probe conductivity measurement on single crystals of these MOFs shows values of 3.95 × 10^–6^, 1.49 × 10^–5^, 8.64 × 10^–5^, and 2.86 × 10^–4^ S cm^–1^ for M = Zn, Co, Mn, and Cd, respectively, and semiconducting behavior. The cation radius determines the S···S distance between neighboring TTF cores and, therefore, the intermolecular interactions and, thus, the conductivity of the MOFs. The shorter the S···S distance, the higher the electrical conductivity. In this family, the S···S distance is inversely proportional to the ionic radius of the metal ions. Larger cations increase the chemical pressure and produce a contraction of the S···S distance that results in better orbital overlap between the p_*z*_ orbitals of neighboring atoms and in an increase of the electrical conductivity. Accordingly, the conductivity values follow the same order as the cation radii and the electrical conductivities can thus be tuned by tuning the cation size of this class of MOFs with π-stacked motifs.^109^

#### 2.1.6 π···π Stacking Interactions

**Scheme 4:**
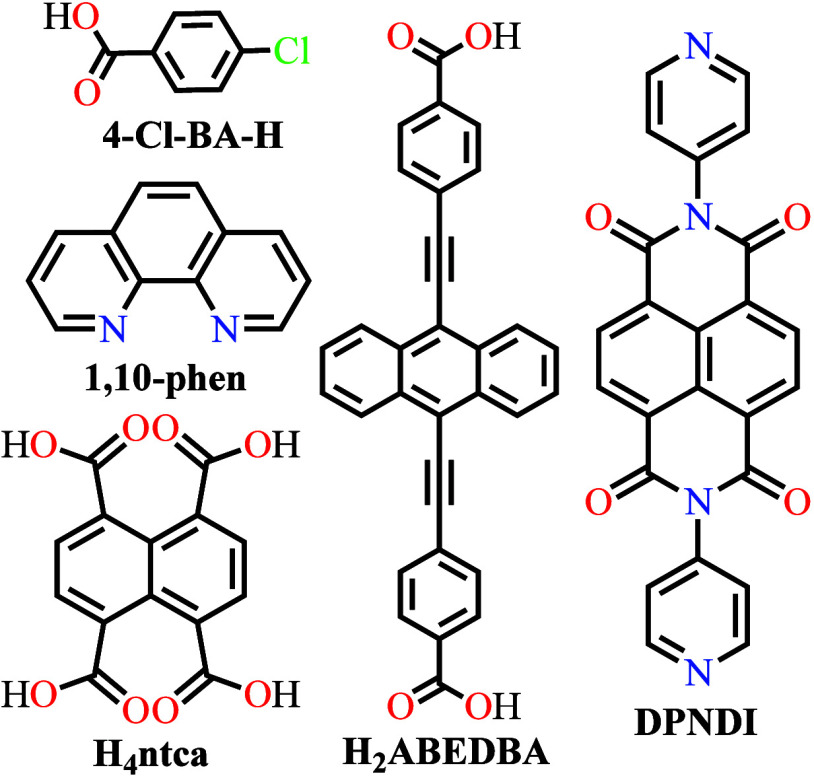
Aromatic Ligands Used for the Design of Conducting MOFs Using π···π Interactions

Planar π-conjugated organic moieties can form π···π interactions within the frameworks, giving rise to electrical conducting networks (Table 8). Based on this idea, different organic ligands like anthracene, naphthalene, phenanthroline, etc., have been used to design conducting MOFs through interchain, interlayer, or intraframework π···π interactions. Recently, Dubey and co-workers have reported the semiconducting behavior of the 1D coordination polymer [Cu_2_(4-ClBA)_4_(4,4′-bipy)]·DMF (**57**) (4-ClBA = 4-chlorobenzoate and 4,4′-bipy = 4,4′-bipyridine, Scheme 4) that shows interchain π···π interactions to form an interpenetrated 3D supramolecular structure.^110^ The distance between the π-assembled 4-ClBA ligands is 3.92 Å. Two-probe conductivity measurement shows that the electrical conductivity of this MOF is 2.8 × 10^–6^ S cm^–1^, which increases to 3.9 × 10^–6^ S cm^–1^ upon desolvation (**57-des**). The authors suggest that the desolvation may increase the extended conjugation within the coordination chains. The same group has also reported the semiconducting 2D MOF [Cu(ndc)(1,10-phen)] (**58**) (ndc = 2,6-napthalenedicarboxylate and 1,10-phen = 1,10-phenanthroline, Scheme 4). This MOF presents interlayer π···π interactions and forms an interpenetrated 3D supramolecular network.^60^ The average distance between the aromatic rings is ∼3.50 Å. This MOF is also semiconducting with a room temperature conductivity of 3 × 10^–3^ S cm^–1^ that increases 16 times on decomposition of the framework to CuO-NPs.

**Table 8:** Conducting MOFs Based on π···π Interactions

#	Complex	Ligand	Dim	*d* (Å)^a^	σ (S cm^–1^)	ref
**57**	[Cu_2_(4-ClBA)_4_(4,4′-bipy)]•DMF	4-Cl-BA-H	1D	3.92	2.8 × 10^–6^^b^	(110)
**57-des**	[Cu_2_(4-ClBA)_4_(4,4′-bipy)]	4-Cl-BA-H	1D	–	3.9 × 10^–6^^b^	(110)
**58**	[Cu(ndc)(1,10-phen)]	1,10-phen	2D	3.50	3 × 10^–3^^b^	(60)
**59**	[ZnNa_2_(ABEDBA)_2_(DEF)_2_]·DEF	H_2_ABEDBA	3D	3.4	1.3 × 10^–3^^c^	(111)
**60**	[Cd(DPNDI)(OH_2_)_4_](NO_3_)_1.3_·*n*DMA	DPNDI	3D	3.18	3.3 × 10^–3^^c^	(112)
**60-des**	Cd(DPNDI)(OH_2_)_4_](NO_3_)_1.3_	DPNDI	3D	–	3.7 × 10^–2^^c^	(112)
**61**	Cu(DPNDI)(PF_6_)_2_(DMA)_4_(CH_3_CN)	DPNDI	3D	3.7	1.2 × 10^–5^^b^	(113)
**62**	[Sr-(ntca)(H_2_O)_2_]·H_2_O	H_4_ntca	2D	3.4	10^–4^^c^	(114)

a Distance between neighboring π-ligands.

b Four-probe on pressed pellet.

c Two-probe on single crystal.

Chen and co-workers used the anthracene-based organic ligand H_2_ABEDBA (4,4′-(anthracene-9,10-diylbis(ethyne-2,1-diyl))dibenzoic acid, Scheme 4) to synthesize a double wall-based 3D MOF [ZnNa_2_(ABEDBA)_2_(DEF)_2_]·DEF (**59**).^111^ The π···π interaction operative between the anthracene moieties provides a 1D long-range conjugated pathway for charge transport. The distance between two neighboring anthracene moieties is 3.4 Å. Electrical conductivity measurements on single crystals of this MOF show a semiconducting behavior with a conductivity value of 1.3 × 10^–3^ S cm^–1^ at 300 K.

**Figure 16:**
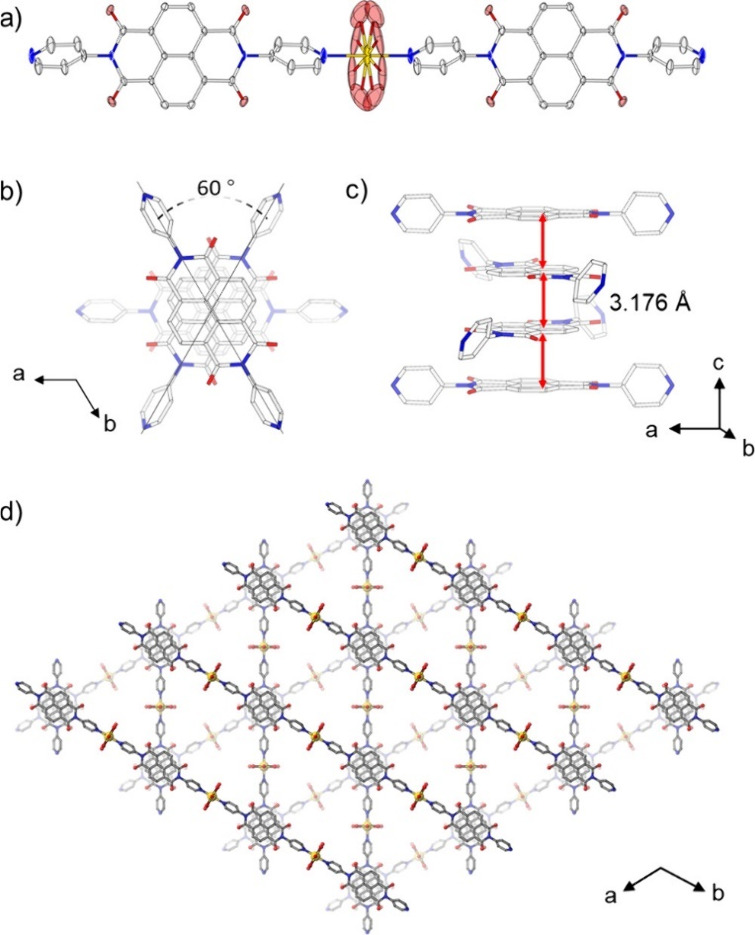
Crystal structure of [Cd(DPNDI)(OH_2_)_4_](NO_3_)_1.3_·*n*DMA (60) (space group *P*6_2_22). (a) Thermal ellipsoids plot of the linear CP in [Cd(DPNDI)(OH_2_)_4_](NO_3_)_1.3_·*n*DMA. (b) The π-stacked DPNDI column projected along the *c*-axis. (c) The columnar structure of DPNDI showing the interplanar distances. (d) Perspective view of [Cd(DPNDI)(OH_2_)_4_](NO_3_)_1.3_·*n*DMA along the *c*-axis. Color code: Cd = yellow, N = blue, C = gray, and O = red. Reproduced from ref (112). Copyright 2019 American Chemical Society.

Hu et al. designed the porous conducting polymer [Cd(DPNDI)(OH_2_)_4_](NO_3_)_1.3_·*n*DMA (**60**) using *N,N*′-di(4-pyridyl)-1,4,5,8-naphthalenetetracarboxdiimide (DPNDI) by an electro-crystallization method.^112^ [Cd(OH_2_)_4_] units are connected by DPNDI ligands to form 1D coordination polymers which are stacked by interchain π···π interactions to form a 3D supramolecular network with hexagonal channels. The interplanar distance between the aromatic groups of the DPNDI moieties is 3.18 Å (Figure 16). Electrical conductivity measurement on single crystals shows conductivity values ranging between 1.0 × 10^–3^ to 3.3 × 10^–3^ S cm^–1^ at 300 K, much higher than the conductivity value measured on pressed pellets. Upon removal of the solvent molecules (**60-des**), the conductivity of the sample increases to 1.2–3.7 × 10^–2^ S cm^–1^.

Kuang et al. reported a similar 3D porous MOF: [Cu(DPNDI)(PF_6_)_2_(DMA)_4_(CH_3_CN)] (**61**) prepared by a slow diffusion method. This 3D porous MOF is formed by π···π interactions among the DPNDI moieties to form an interpenetrated structure.^113^ The distance between the π-assembled DPNDI moieties is 3.7 Å. Single crystal electrical conductivity measurements show a room temperature value of 1.2 × 10^–5^ S cm^–1^. Haider and co-workers synthesized another 2D MOF [Sr(ntca)(H_2_O)_2_]·H_2_O (**62**) (ntca = 1,4,5,8-naphthalenetetracarboxylic acid).^114^ Within the 2D layers, the ntca ligands are connected by π···π interactions, and the distance is ∼3.4 Å. The electrical conductivity at room temperature of the material is 10^–4^ S cm^–1^.

### 2.2 Extrinsically Conducting MOFs

In extrinsically conducting MOFs, the electrical conductivity is strongly dependent on guest molecules, incorporated either through in situ methods or by post-synthetic modifications within their pores. These guest molecules can be classified as (a) metal-based guests, (b) organic, inorganic and organometallic guest molecules and (c) organic conducting polymers. In the following section, we will show the most important examples of extrinsically conducting MOFs prepared with different types of guests.

#### 2.2.1 Metal-Based Guest

**Figure 17:**
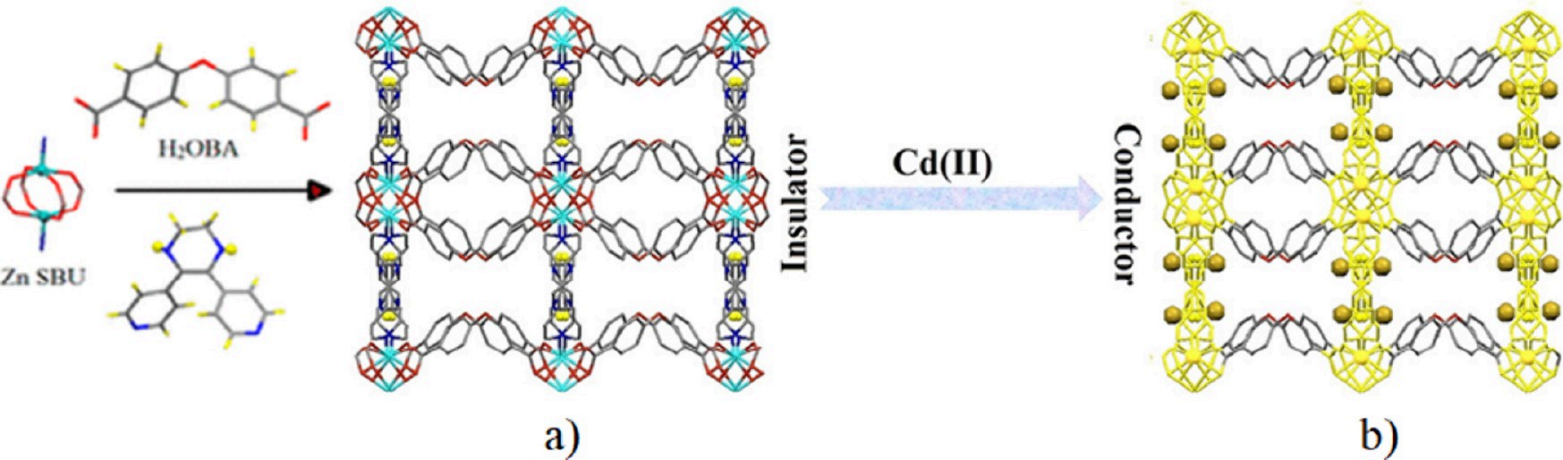
(a) View of the 3D framework of MOF [Zn(OBA)(DPTHD)]·DMF (**63**). (b) Possible pathway for electron transfer (yellow) for Cd@MOF (golden spheres are the Cd^2+^ ions). Color code: C = gray, S = yellow, Zn = cyan, N = blue, and O = red. Reproduced from ref (115). Copyright 2019 American Chemical Society.

The insertion of metal atoms and metallic nanoparticles has been used by different research groups as a strategy to increase the electrical conductivity of porous MOFs. Morsali and co-workers have shown the possibility to modulate the electrical conductivity of the amine-functionalized MOF [Zn(OBA)(DPTHD)]·DMF (**63**), where H_2_OBA = 4,4′-oxybisbenzoic acid and DPTHD = 5,6-di(pyridin-4-yl)-1,2,3,4-tetrahydropyrazine, by incorporation of Cd^2+^ ions. (Figure 17). The encapsulation of Cd^2+^ ions within this framework increases the electrical conductivity from 5.8 × 10^–6^ S cm^–1^ to 1.8 × 10^–2^ S cm^–1^.^115^

**Figure 18:**
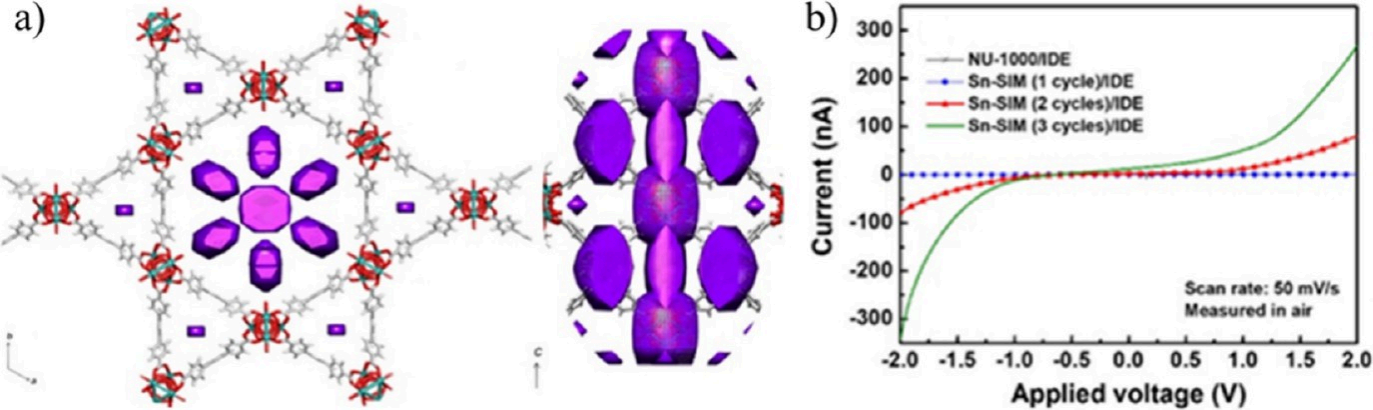
(a) SnO_2_@NU-1000 (3 cycles). The electron density of SnO_2_ is presented in purple. (b) I–V curves of NU-1000/IDE, SnO_2_@NU-1000 (1 cycle)/IDE, SnO_2_@NU-1000 (2 cycles)/IDE, and SnO_2_@NU-1000 (3 cycles)/IDE, measured in air at room temperature. Color code: C = gray, Zr = green, and O = red. Reproduced from ref (117). Copyright 2018 American Chemical Society.

Several groups have used metal nanoparticles (NPs) as the guest species in order to increase the electrical conductivity of different MOFs (Table 9). Thus, Han et al. synthesized a semiconducting MOF through encapsulation of AgNPs within the insulating framework.^116^ A cyclodextrin-based Rb-CD-MOF (**64**) adsorbs Ag^+^ ions upon soaking the MOF crystals in AgNO_3_ solutions. The adsorbed Ag^+^ ions are reduced by the hydroxide groups of the cyclodextrin moieties to form AgNPs. Such encapsulation of AgNPs within the MOF increases the electrical conductivity to 3.1 × 10^–9^ S cm^–1^. Kung and co-workers successfully enhanced electrical conductivity of NU-1000 MOF through incorporation of SnO_2_ within the pores.^117^ SnO_2_ was deposited within pores of the MOF by consequent solution and vapor phase treatment of Sn(amd)_2_, where amd = bis(N,N′-di-*i*-propylacetamidinato) (Figure 18). After three consecutive cycles of incorporation, the obtained SnO_2_@NU-1000 MOF (**65**) shows a conductivity value of ∼1.8 × 10^–7^ S cm^–1^, while the parent MOF is an insulator. The formation of SnO_2_ pillars within the void space of MOF acts as the charge transport pathway within the framework.

**Table 9:** Conducting MOFs with Metal-Based Guests

#	MOF	Guest	Dim	σ_MOF_ (S cm^–1^)	σ_Guest@MOF_ (S cm^–1^)	ref
**63**	[Zn(OBA)(DPTHD)]·DMF	Cd^2+^	3D	5.8 × 10^–6^^a^	1.8 × 10^–2^^a^	(115)
**64**	Rb-CD-MOF	AgNPs	3D	6.8 × 10^–10^^b^	3.1 × 10^–9^^b^	(116)
**65**	NU-1000	SnO_2_	3D	≤10^–12^^c^	1.8 × 10^–7^^c^	(117)

a Impedance on pressed pellet.

b Two-probe on single crystal.

c Two-probe on thin film.

#### 2.2.2 Molecular Guests

Different types of electroactive molecules and molecular entities, including tetrathiafulvalene, TCNQ (7,7,8,8-tetracyanoquinodimethane), iodine, NiCB, methyl viologen, etc., have been encapsulated within the channels of MOFs to induce/enhance the electrical conductivity of the parent MOFs.^118−120^

**Figure 19:**
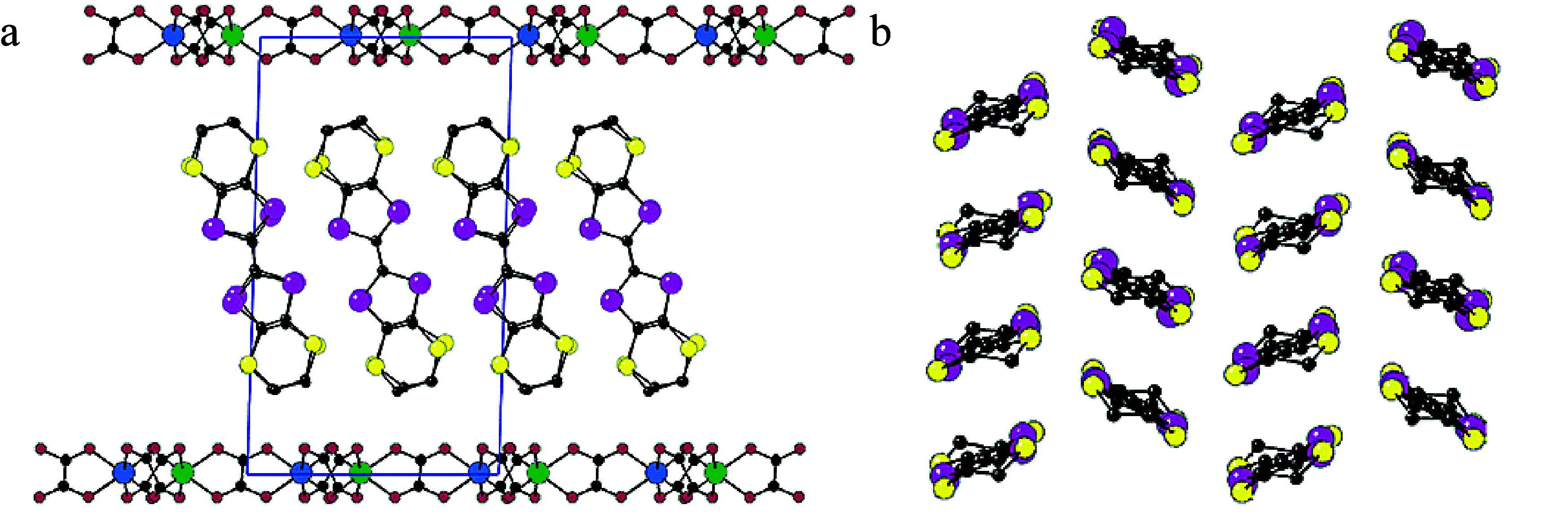
(a) Side view of the alternating layers in [BEDT-TSF]_*x*3_[MnCr(C_2_O_4_)_3_]·(CH_2_Cl_2_) (**67**). (b) Top view of the organic layer. Color code: C = gray, S = yellow, Se = pink, Mn = blue, Cr = green, and O = red. Reproduced from ref (36). Copyright 2003 American Chemical Society.

In 2000, Coronado, Gómez-García et al. encapsulated BEDT-TTF molecules within the interlamellar space of a 2D magnetically ordered framework by an in situ electrocrystallization synthetic method. Within the host–guest (BEDT-TTF)_3_[MnCr(C_2_O_4_)_3_] (**66**) (C_2_O_4_^2–^ = oxalate dianion), the BEDT-TTF cations form pseudohexagonal β-type layers intercalated within honeycomb 2D coordination layers of [MnCr(C_2_O_4_)_3_]^−^. Four-probe electrical conductivity measurements within the plane showed a very high room temperature conductivity value of 250 S cm^–1^ and a metallic conductivity within the temperature range 2–300 K.^35^ Furthermore, the anionic honeycomb layers display a long-range ferromagnetic order at low temperatures. In another report, the same group synthesized a similar type of framework with the selenium-substituted organic donor bis(ethylenedithio)-tetraselanafulvalene (BEDT-TSF): (BEDT-TSF)_3_[MnCr(C_2_O_4_)_3_] (**67**) (Figure 19).^36^ As in the BEDT-TTF compound, the BEDT-TSF moieties are located within the interlamellar space of 2D honeycomb layers of [MnCr(C_2_O_4_)_3_]^−^, and the average charge on each BEDT-TSF moiety is 1/3. The framework also shows a ferromagnetic long-range order at low temperatures. The room temperature electrical conductivity of the Se-derivative is 1 S cm^–1^, and it shows metallic conductivity within the range of 150–300 K.

The same group also prepared the host–guest charge transfer complex formulated as (BEDT-TTF)_*x*_[MnRh(C_2_O_4_)_3_]·CH_2_Cl_2_ (**68**) (where *x* = 2.526(1)) by replacing Cr with Rh. The room-temperature electrical conductivity of this compound is 13 S cm^–1^ and shows a metallic behavior, reaching a value of 28 S cm^–1^ at 103 K.^37^

**Figure 20:**
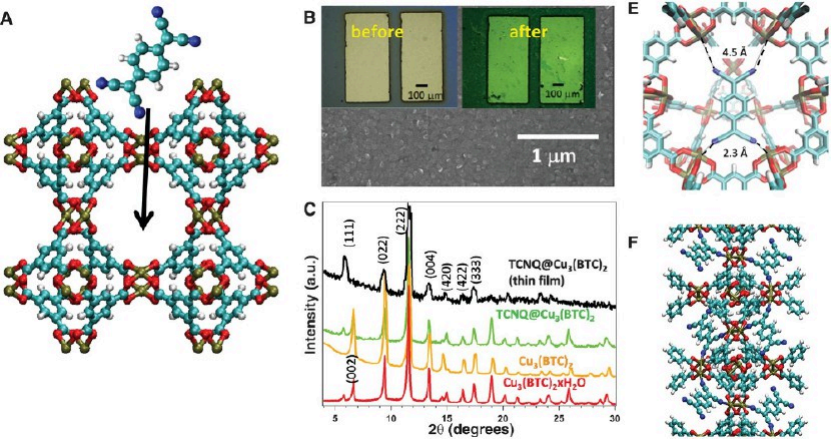
(a) TCNQ molecule shown above a Cu_3_(BTC)_2_ MOF; arrow points into the pore. Color code: H = white, N = blue, C = cyan, O = red, and Cu = light brown. (b) SEM image of a MOF-coated device; insets are optical images of devices before and after TCNQ infiltration. (c) XRD data for powders and grazing incidence XRD for a thin film. (e) Minimum-energy configuration for TCNQ@Cu_3_(BTC)_2_ obtained from ab initio calculations. (f) Possible configuration that would provide a conductive channel through the MOF unit cell. Color code: C = cyan, Cu = yellow, and O = red. Reproduced with permission from ref (122). Copyright 2013 The American Association for the Advancement of Science.

The electrical conductivity of MOFs having nanopores can be tuned by filling them up with redox-active conjugated guest molecules such as 7,7,8,8-tetracyanoquinodimethane (TCNQ).^121^ Thus, Allendorf et al. encapsulated TCNQ within a thin film of MOF Cu_3_(BTC)_2_ (HKUST-1, **69**), where H_3_BTC = benzene-1,3,5-tricarboxylic acid, by soaking the MOF in a saturated solution of TCNQ.^122^ Spectroscopic and structural analysis shows a coordination between the Cu^2+^ centers and TCNQ (Figure 20). Such encapsulation increases the electrical conductivity of the framework from 10^–8^ S cm^–1^ to 7 × 10^–2^ S cm^–1^ at room temperature. Later, Allendorf, Fischer, and co-workers synthesized TCNQ-incorporated HKUST-1 with the precise molecular formula TCNQ_1.0_@Cu_3_BTC_2_ that shows an electrical conductivity value of 1.5 × 10^–4^ S cm^–1^.^123^ Thürmer et al. have also prepared thin films of HKUST-1 on ITO, and then TCNQ was inserted within the pores of the MOF. The resultant TCNQ@HKUST-1 has been found to be 10^10^ times more conductive (10^–1^ S cm^–1^) than the parent MOF (10^–11^ S cm^–1^).^124^ In another report, Deep et al. prepared thin films of TCNQ@HKUST-1 on a gold-screen printed electrode through a prior thin film fabrication of the MOF and found an increase of the electrical conductivity of 10^9^ times.^125^ Vittal, Loh, and co-workers developed a thin film (thickness ∼70 nm) of the 2D MOF [Cu_2_(AcO)_4_(CuTPyP)_0.5_]·CHCl_3_ (**70**) (H_2_TPyP = 5,10,15,20-tetra-4-pyridyl-21H,23H-porphine) with inserted TCNQ. The resultant TCNQ@2D-MOF shows a conductivity of 10^–6^ S cm^–1^, which is 10^3^ times higher than that of the parent MOF (10^–9^ S cm^–1^).^126^

**Figure 21:**
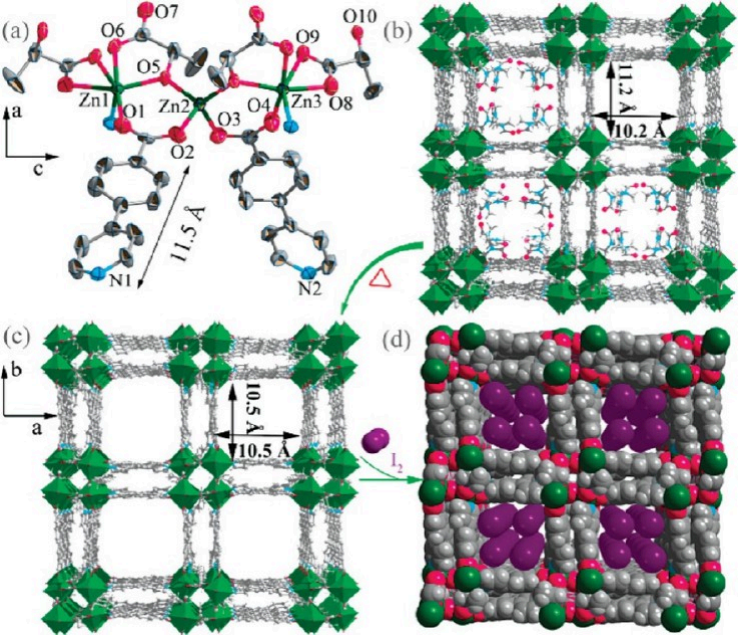
(a) Coordination environment of Zn atoms in [Zn_3_(d,l-lac)_2_(pybz)_2_]·2.5DMF (H-atoms are omitted for clarity). (b) Perspective views of the 3D open framework with 1D channels in [Zn_3_(d,l-lac)_2_(pybz)_2_]·2.5DMF with the guest DMF molecules. Color code: Zn = green, N = blue, O = red, and C = gray. (c) The completely desolvated framework of [Zn_3_(d,l-lac)_2_(pybz)_2_]·2.5DMF, showing the empty channels. (d) Location of the I_2_ molecules in the channels of desolvated framework of [Zn_3_(d,l-lac)_2_(pybz)_2_]·2.5DMF. The PLATON calculated void space in the desolvated framework of [Zn_3_(d,l-lac)_2_(pybz)_2_]·2.5DMF is 43.8%. Reproduced from ref (128). Copyright 2010 American Chemical Society.

Incorporation of iodine and polyiodides within the channels of MOFs is one of the easiest and most attractive strategies to enhance the electrical conductivity of MOFs. Due to its large size, electron density, and low electronegativity, I_2_ becomes an ideal dopant in the form of either vapor or solution or even as a template.^127^ Zeng et al. loaded I_2_ within the 1D channels (∼11 × 10 Å^2^) of MOF [Zn_3_(d,l-lac)_2_(pybz)_2_]·2.5DMF (**71**) (where d,l-lac = lactate and pybz = 4-pyridine benzoate anions) by soaking the MOF in a cyclohexane solution of I_2_ (Figure 21). The electrical conductivity of I_2_@MOF is 3.42 × 10^–3^ S cm^–1^ along the channels and 1.65 × 10^–4^ S cm^–1^ in the direction perpendicular to the channels. Both values are much higher than those of the pristine MOF (7 × 10^–6^ S cm^–1^).^128^

In another report, Zeng et al. showed that the incorporation of I_2_ within the channels of [Co^II^_3_(lac)_2_(pybz)_2_]·3DMF (**72**) (where pybz = 4-pyridyl benzoate and lac = d- and l-lactate) modifies the electrical conductivity. The host–guest MOF [Co^II^_3_(pybz)_2_(lac)_2_]·2.7I_2_ shows a semiconducting behavior with a room-temperature conductivity value of 7 × 10^–6^ S cm^–1^.^129^ Kobayashi et al. showed the modulation of the electrical conductivity of a redox-active dithiolate-based MOF Cu[Ni(pdt)_2_] (**73**) (CuNi) (pdt^2–^ = pyrazine-2,3-dithiolate) through encapsulation of I_2_ vapor within the channels. The resultant I_2_@CuNi shows 10^4^ times higher conductivity (10^–4^ S cm^–1^ with an activation energy of 490 meV) than the parent CuNi MOF (10^–8^ S cm^–1^).^130^ Yin et al. synthesized an I_2_-doped MOF [Cu_6_(pybz)_8_(OH)_2_]·(I_5_)·(I_7_) (**74**) by using iodine as a precursor template and transformed it into [Cu_6_(pybz)_8_(OH)_2_](I)_2_·3.5CH_3_OH (**75**) by soaking the crystals in methanol. The former compound with polyiodides shows a 100 times higher conductivity (8.11 × 10^–7^ S cm^–1^) than the later one with I^–^ (8.04 × 10^–9^ S cm^–1^).^131^

**Figure 22:**
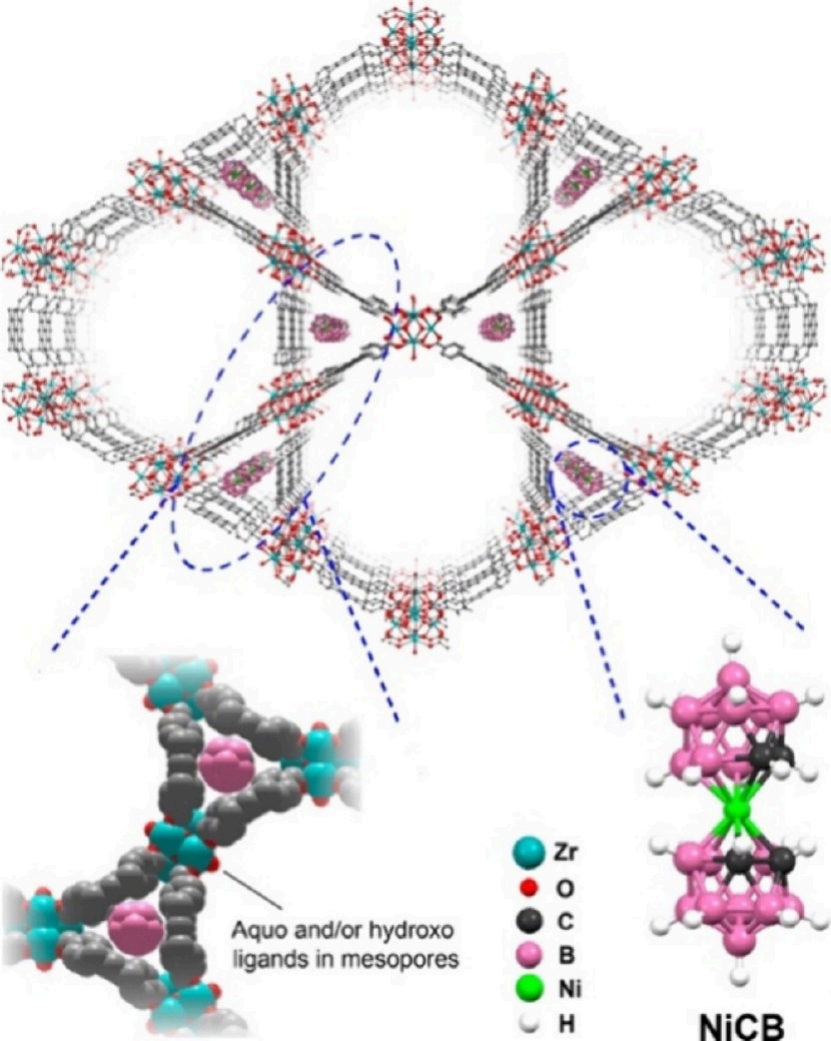
Crystal structure of NiCB@NU-1000. The triangular channels of NiCB@NU-1000 are shown in a space-filling model to present the close interaction between NiCB and three surrounding pyrenes. H-atoms have been omitted for clarity. The structure of NiCB is also presented. Reproduced from ref (132). Copyright 2018 American Chemical Society.

Kung and co-workers modified the electrical conductivity of the porous NU-1000 MOF [Zr_6_(μ^3^-OH)_8_(OH)_8_(TBAPy)_2_] (**76**) (H_4_TBAPy = 1,3,6,8-tetrakis(*p*-benzoic acid)pyrene) by introducing electron-deficient [Ni(dicarbollide)_2_] (NiCB) molecules in the pores of the framework containing an electron-rich pyrene core as the ligand backbone (Figure 22).

**Figure 23:**
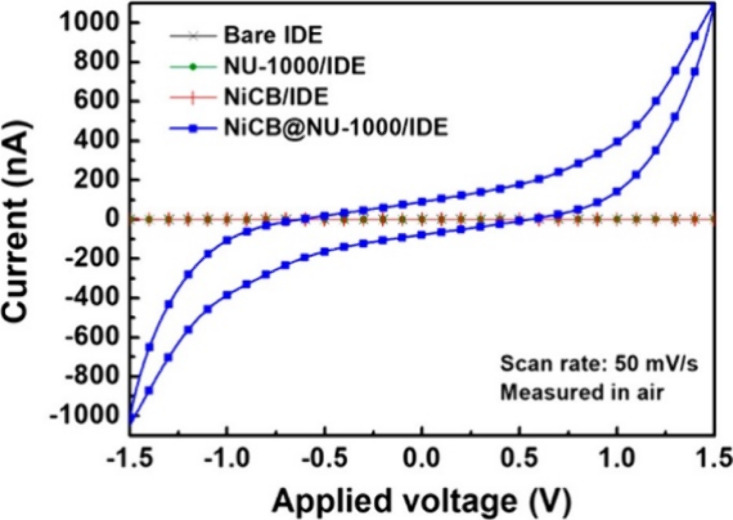
I–V curves of the bare IDE, NU-1000/IDE, NiCB/IDE, and NiCB@NU-1000/IDE. Reproduced from ref (132). Copyright 2018 American Chemical Society.

Single crystal X-ray structural analysis reveals that NiCB molecules are loaded within the narrow triangular channels but not in the large hexagonal channels. Both NiCB and parent MOF are insulating, whereas the conductivity of NiCB@MOF (**76**) is 2.7 × 10^–7^ S cm^–1^ (Figure 23).^132^ Such enhancement of electrical conductivity is due to donor–acceptor charge transfer between NiCB and the pyrene-based organic ligand of the framework.

Guo et al. modulated the electrical conductivity of a blue pillared-paddle-wheel MOF [Zn_2_(TCPB)(BPDPNDI)] (**77**) (BPDPNDI = *N,N*′-bis(4-pyridyl)-2,6-dipyrrolidyl naphthalenediimide and TCPB = 1,2,4,5-tetrakis(4-carboxy phenyl)benzene) by introducing different organic moieties such as MV^2+^, 1,5-difluoro-2,4-dinitrobenzene (DFDNB), dinitrotoluene (DNT), and C_60_ within the pores.^133^ The electrical conductivity was measured on thin films of the samples. Interestingly, MV^2+^@MOF shows a 35 times enhancement of the conductivity value (2.3 × 10^–5^ S cm^–1^) compared to the parent MOF (6 × 10^–7^ S cm^–1^). In DFDNB@MOF (3.5 × 10^–6^ S cm^–1^) and DNT@MOF (1.5 × 10^–6^ S cm^–1^), the conductivity values also show an important increment of 1 order of magnitude. In contrast, C_60_@MOF (4 × 10^–7^ S cm^–1^) does not show any change (Table 10).

**Table 10:** Conducting MOFs with Molecular Guests

#	MOF	Guest	Dim	σ_MOF_(S cm^–1^)	σ_guest@MOF_(S cm^–1^)	ref
**66**	(BEDT-TTF)_3_[MnCr(oxalate)_3_]	BEDT-TTF	3D	–	250^a^	(35)
**67**	(BEDT-TSF)_3_[MnCr(oxalate)_3_]·CH_2_Cl_2_	BEDT-TSF	3D	–	1–23^a^	(36)
**68**	(BEDT-TTF)_*x*_ [MnRh(oxalate)_3_]·CH_2_Cl_2_	BEDT-TTF	3D	–	13^b^	(37)
**69**	HKUST-1	TCNQ	3D	10^–8^^g^	7 × 10^–2^^g^	(122)
**70**	[Cu_2_(AcO)_4_(CuTPyP)_0.5_]·CHCl_3_	TCNQ	3D	10^–9^^c^	10^–6^^c^	(126)
**71**	[Zn_3_(d,l-lac)_2_(pybz)_2_]·2.5DMF	I_2_	3D	–	3.42 × 10^–3^^a^	(128)
**72**	[Co^II^_3_(lac)_2_(pybz)_2_]·2.7 I_2_	I_2_	3D	–	7 × 10^–6^^d^	(129)
**73**	Cu[Ni(pdt)_2_]	I_2_	3D	10^–8^^e^	10^–4^^e^	(130)
**74**	[Cu_6_(pybz)_8_(OH)_2_]·(I_5_)·(I_7_)	I_5_^–^ + I_7_^–^	3D	–	8.11 × 10^–7^^g^	(131)
**75**	[Cu_6_(pybz)_8_(OH)_2_](I)_2_·3.5CH_3_OH	I^–^	3D	–	8.04 × 10^–9^^f^	(131)
**76**	[Zr_6_(μ_3_-OH)_8_(OH)_8_(TBAPy)_2_]	[Ni(dicarbollide)_2_]	3D	Insulator^d^	2.7 × 10^–7^^d^	(132)
**77**	[Zn_2_(TCPB)(BPDPNDI)]	MV^2+^	3D	6 × 10^–7^^e^	2.3 × 10^–5^^e^	(133)
**77**	[Zn_2_(TCPB)(BPDPNDI)]	DFDNB	3D	6 × 10^–7^^e^	3.5 × 10^–6^^e^	(133)
**77**	[Zn_2_(TCPB)(BPDPNDI)]	DNT	3D	6 × 10^–7^^e^	1.5 × 10^–6^^e^	(133)
**77**	[Zn_2_(TCPB)(BPDPNDI)]	C_60_	3D	6 × 10^–7^^e^	4 × 10^–7^^e^	(133)

a Two-probe on single crystal.

b Four-probe on single crystal.

c Two-probe on thin film.

d Two-probe on pressed pellet.

e Four-probe on thin film.

f Four-probe on pressed pellet.

g Thin film.

#### 2.2.3 Organic Conducting Polymers Guests

The insertion of organic conducting polymers like PEDOT (polymer of 3,4-ethylenedioxythiophene),^134^ polyaniline (PANI),^135^ and polypyrrole (PPy)^136^ within the channels of MOFs has also been used in order to combine the novel functionalities of MOFs with the useful properties of conducting polymers.

**Figure 24:**
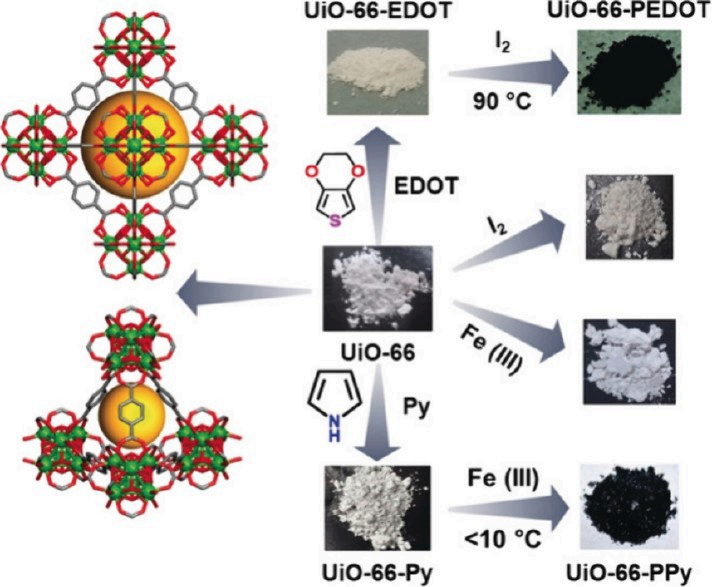
Reaction scheme for the synthesis of PEDOT@UiO-66 and Ppy@UiO-66. Left panel: Visualizing two different pores in UiO-66 (color code: Zr = green, O = red, and C = gray; H atoms are omitted for clarity. Right panel: Optical images of products obtained after each step. Reproduced with permission from ref (139). Copyright 2019 John Wiley and Sons.

Although not within a MOF, as far as we know, the first attempts to insert a conducting polymer within a porous material were performed by inserting PEDOT inside porous NaX and NaY faujasites.^137^ Conductivity measurements on thin films of this material show a huge increase in the conductivity of PEDOT@NaY with a value similar to that found in bulk PEDOT and much higher than that measured in the pristine NaY faujasite. Following the same strategy, Kitagawa and co-workers synthesized PEDOT encapsulated within the void space of MIL-101(Cr) (**78**) by using the monomer 3,4-ethylenedioxythiophene (EDOT) following a two-step method: adsorption of monomer and then I_2_-mediated oxidative polymerization. These authors observed that PEDOT@MIL-101(Cr) has 10^8^ times higher conductivity (1.1 × 10^–3^ S cm^–1^) in the presence of I_2_ as a dopant than the parent MOF (∼10^–11^ S cm^–1^). They also found that the conductivity of PEDOT@MIL-101(Cr) varies with the amount of polymer present within the channels.^138^ Ballav and co-workers have modulated the insulating behavior of the biporous UiO-66 (**79**) MOF by incorporating two different types of conducting polymers (PEDOT and PPy) within the pores in a similar two-step method (Figure 24). Four-probe electrical conductivity measurements show room-temperature electrical conductivity values of 2 × 10^–2^ S cm^–1^ and 10^–3^ S cm^–1^ for UiO-66-PPy and UiO-66-PEDOT, respectively, while UiO-66 (**79**) is insulating, and the electrical conductivities of the physical mixtures of UiO-66 (**79**) with the bulk polymers vary between 10^–6^ and 10^–7^ S cm^–1^.^139^

**Figure 25:**
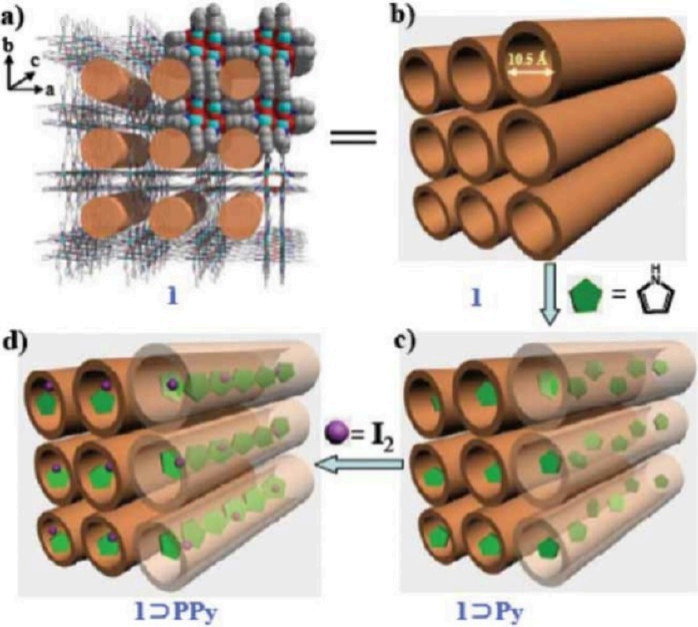
Schematic illustration of the formation of PPy in the channels of [Zn_3_(d,l-lactate)_2_(4-pyridylbenzoate)_2_] (**80**). (a) 3D open framework with 1 nm nanochannels. (b) Empty framework of **80**. (c) Encapsulation of Py in the channels of **80**. (d) Polymerization of Py with iodine in **80**. Color code: C = gray, Zn = cyan, N = blue, and O = red. Reproduced with permission from ref (140). Copyright 2011 John Wiley and Sons.

Wang and co-workers observed the enhancement of the electrical conductivity of a porous MOF upon incorporation of conducting PPy chains within the nanochannels. They prepared polymeric chains of pyrrole (PPy) within the 1D channels of MOF [Zn_3_(d,l-lactate)_2_(4-pyridylbenzoate)_2_] (**80**) (Figure 25) via oxidative polymerization in the presence of 0.05 M I_2_. Conductivity measurements show that PPy@MOF has 10^5^ times higher conductivity (10^–2^ S cm^–1^) than bulk PPy (10^–7^ S cm^–1^) and 10 times higher conductivity than I_2_@MOF.^140^ The encapsulated PPy acts as the main charge transport pathway.

Similarly, Dhara et al. prepared PPy@Cd-MOF (Cd-MOF = [Cd_2_(NDC)(PCA)_2_]·G_*x*_ (**81**) with H_2_NDC = 2,6-napthalenedicarboxylicacid, HPCA = 4-pyridinecarboxylic acid, and G = guest molecules) with a similar oxidative polymerization method. PPy@Cd-MOF shows 10^9^ times higher conductivity (10^–3^ S cm^–1^) than the parent MOF (10^–12^ S cm^–1^) and 10^4^ times higher than I_2_@Cd-MOF (10^–7^ S cm^–1^).^141^

**Figure 26:**
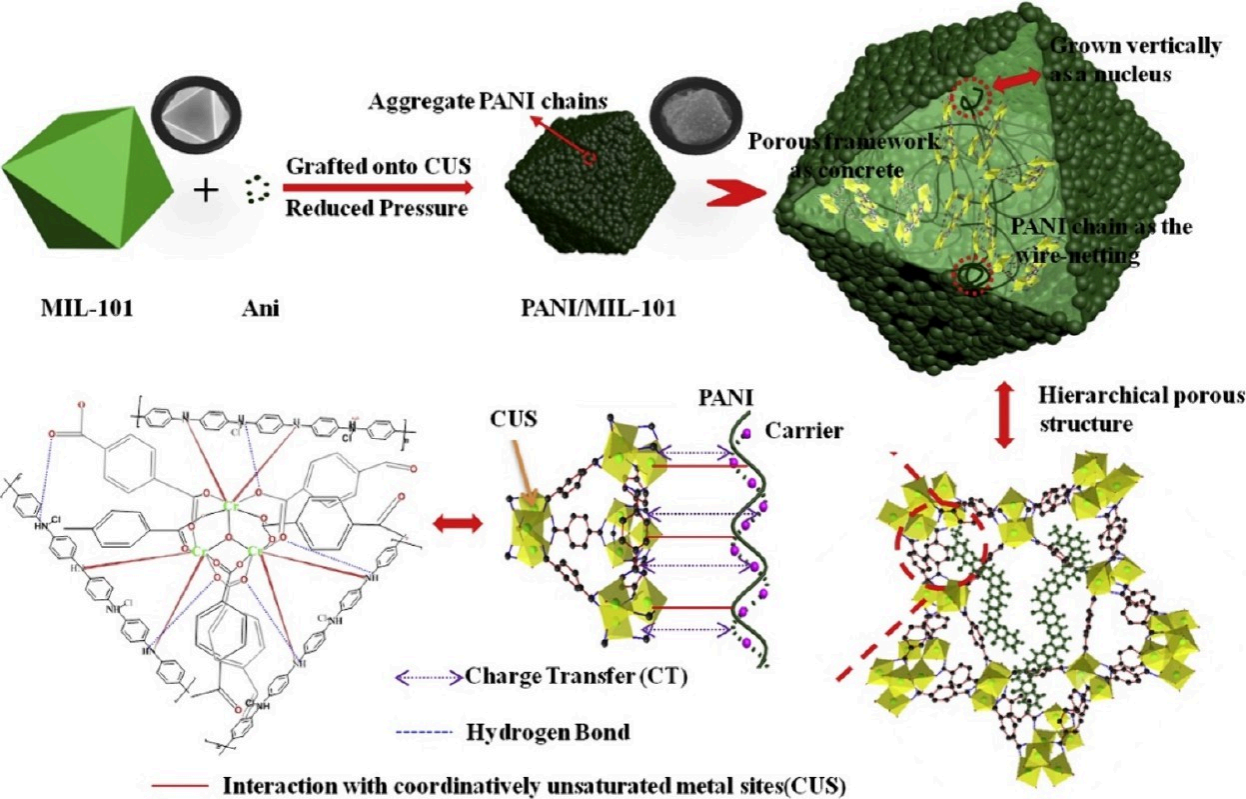
Schematic illustration of the fabrication process and the interaction between PANI and MIL-101(Cr). Reproduced with permission from ref (142). Copyright 2018 Elsevier.

Shao et al. developed a composite material of polyaniline (PANI) and MIL-101(Cr) (**78**) in which PANI moieties are present within the channels as well as at the surface of the framework (Figure 26). The encapsulated PANI moieties act as the charge transport media throughout the framework.

Aniline molecules were adsorbed within the pores of MIL-101(Cr) (**78**) under reduced pressure and then polymerized. At low concentrations, the polymers are formed within the pores, while at high concentrations, the size of the polymer chains becomes larger and the polymers cover the surface. Electrical conductivity measurements show that increasing the concentration of PANI within the MOF pores leads to higher conductivity values with a maximum conductivity of 0.55 S cm^–1^ for a 20% loading and a decrease for higher PANI loadings (Table 11).^142^ Shao and co-workers also loaded PANI within the pores of UiO-66 MOF (**79**) under similar conditions. The incorporation of PANI within UiO-66 MOF (**79**) was verified by XRPD, IR, UV–vis, SEM, and gas adsorption analyses. The electrical conductivity of PANI@UiO-66 (≈0.2 S cm^–1^) is higher than that of PANI itself, while the MOF is an insulator.^143^

**Table 11:** Conducting MOFs with Conducting Polymer Guests

#	MOF	Guest	Dim	σ_MOF_ (S cm^–1^)	σ_Guest@MOF_ (S cm^–1^)	ref
**78**	MIL-101(Cr)	PEDOT + I_2_	3D	10^–11^^a^	1.1 × 10^–3^^a^	(138)
**78**	MIL-101(Cr)	20% PANI	3D	10^–11^^b^	0.55^b^	(142)
**79**	UiO-66	PEDOT	3D	Insulator^b^	10^–3^^b^	(139)
**79**	UiO-66	PPy	3D	Insulator^b^	2 × 10^–2^^b^	(139)
**79**	UiO-66	20% PANI	3D	Insulator^b^	0.2^b^	(143)
**80**	{Zn_3_(d,l-lactate)_2_(4-pyridylbenzoate)_2_}_*n*_	PPy	3D	–	10^–2^^c^	(140)
**81**	[Cd(NDC)_0.5_(PCA)]·G_*x*_	PPy	3D	10^–12^^d^	10^–3^^d^	(141)

a Impedance on pressed pellet.

b Four-probe on pressed pellet.

c Two-probe on single crystal.

d Two-probe on pressed pellet.

## 3 MOF Composites

The combination of MOFs with other conducting carbon-based materials like carbon nanotubes, graphene, graphene oxide, reduced graphene oxide, etc., is another important strategy to improve the bulk electrical conductivity of MOFs as well as their applications (Table 12).^43,44,144^ There are two different ways for such composite formation: physical mixture of MOF with conducting substrates and growth of MOF nanostructures (nano rods, nano wires, nano sheets, etc.) on the conductive substrates.

**Table 12:** Conducting MOF-Composites

#	MOF-composite composition	Dim	σ_MOF_ (S cm^–1^)	σ_MOF-composite_ (S cm^–1^)	ref
**82**	HKUST-1/Polyaniline/Pt + I_2_	3D	Insulator	0.125^a^	(145)
**83**	ZIF-8/RGO (20% wt)	3D	Insulator	0.64^b^	(146)
**84**	ZIF-8/SWCNTs	3D	Insulator	0.056^a^	(147)
**85**	[Co^II^(Co^III^TCPP)]/FTO	3D	–	3.62 × 10^–8^^c^	(148)
**86**	HKUST-1-graphene (40% wt)	3D	Insulator	10^–4^^b^	(149)

a Four-probe on thin film.

b Four-probe on pressed pellet.

c Impedance on thin film.

Lu and co-workers have constructed 3D MOF nanostructures of three different MOFs (HKUST-1, Zn_2_(BDC)_2_DABCO, and MIL-68) on the layers of conducting polyaniline substrates deposited on Pt electrodes.^145^ The obtained composite HKUST-1/polyaniline/Pt (**82**) shows high porosity and large enhancement of electrical conductivity to 0.125 S cm^–1^ (four-probe on thin films) upon I_2_ adsorption, while the HKUST-1 is itself insulating in nature.

Kim et al. have developed a nanocomposite of ZIF-8/graphene (**83**), by templated growth of the MOF nanoparticles on graphene followed by annealing at 650 °C to reduce the graphene oxide.^146^ Such subsequent composite formation and reduction of graphene oxide increase the electrical conductivity as well as the porosity. They have also observed that the conductivity is dependent on the amount of graphene oxide present in the composite. The conductivity value (0.02 S cm^–1^) increased to 0.64 S cm^–1^ with increasing the amount of graphene from 2.5 to 20% wt (measured with the four-probe method on pressed pellets of the composite), while both ZIF-8 and graphene oxide are insulating in nature.

Ellis et al. have reported the controlled growth of ZIF-8 nanoparticles on single-walled carbon nanotubes (SWCNTs). Two-probe electrical conductivity measurement on thin films of the ZIF-8/SWCNTs composite (**84**) shows a large enhancement of the electrical conductivity to 0.056 S cm^–1^.^147^

Ahrenholtz et al. have reported the solvothermal growth of thin films of [Co^II^(Co^III^TCPP)] (**85**) on conducting fluorine-doped tin oxide substrates (FTO) (CoTCPP = [5,10,15,20-(4-carboxyphenyl)porphyrin]cobalt(III)).^148^ The obtained composite shows charge transport through a redox hopping mechanism with a moderate electrical conductivity of 3.62 × 10^–8^ S cm^–1^, as shown by impedance measurement with thin films.

Alfè and co-workers have developed a series of composite of HKUST-1 with different amounts of graphene (**86**).^149^ Four-probe conductivity measurements on pressed pellets show that such composite has an electrical conductivity of 10^–4^ S cm^–1^ (with 40% wt graphene).

## 4 Thin Film Fabrication of MOFs

Thin film fabrication of MOFs is a key step in the development of electronic and optoelectronic technologies.^150,151^ Recently, conductive MOF thin films have gained much attention for electronic applications.^55,152−157^ The methods employed to fabricate thin films of conductive MOFs can be classified into two categories: (a) liquid-phase fabrication and (b) gas-phase fabrication.

In liquid-phase, the main fabrication methods are (i) exfoliation, (ii) drop-cast, (iii) in situ growth from mother solution, (iv) electrochemical deposition, (v) interfacial method, (vi) liquid-phase epitaxy and (vii) layer by layer growth. In gas-phase, the main methods are (i) chemical vapor deposition, (ii) atomic layer deposition and (iii) physical vapor deposition. Herein, we will discuss only those methods which have been more frequently used to fabricate thin films of MOFs.

(a) **Thin film fabrication with presynthesized MOFs.** Ultrasonication-assisted exfoliation of thin films of 2D layered MOFs is an attractive approach. Benmansour et al. have designed highly conductive 2D layered MOFs of mixed-valent Fe-anilates with different halogens in the organic building blocks. Afterward, they exfoliated these 2D MOFs into nanosheets (∼7 nm) by ultrasonication in methanol.^55^ Another approach is drop-casting of the colloidal solution containing microparticles of presynthesized MOFs in the presence/absence of binder. Horiuchi and co-workers have fabricated thin films of MIL-101(Fe) on fluorine-doped tin oxide substrates (FTO) glass substrates with Nafion.^152^
(b) **Layer by layer growth.** In this method, the surface of the conducting substrate is modified by organic building blocks to form self-assembled monolayers (SAM) with free functional groups like −SH, −OH, −NH_2_, −COOH, etc. These free functional groups are reacted with metal ions and then with the organic building blocks to construct MOFs on the SAM layers by alternatively soaking in the solution of metal ions and organic building blocks. The thickness and number of layers in the deposited MOF thin films can be controlled through the repetition of soaking. Using this method, Shekhah et al. have prepared thin films of HKUST-1 on Au substrates modified with 16-mercaptohexadecanoic acid (MHDA) followed by soaking the SAM coated substrate into solutions of copper(II) acetate and H_3_BTC alternatively.^153^
(c) **Interfacial growth method.** In this method, thin films of MOFs are fabricated at the interface of two immiscible solvents. Ameloot et al. have prepared thin films of HKUST-1 by injecting a solution of copper(II)-acetate in water with 4 wt % poly(vinyl alcohol) into a solution of H_3_BTC in octaethanol at the interface of the two solutions.^154^ Bai and co-workers have introduced spray methods to prepare thin films at the liquid–liquid interface. The thin films are prepared by spraying an acetonitrile solution of the metal ion (Cu^2+^) over a DMF solution of BDC^2–^ ligand to form nanosheets of [Cu(BDC)] MOF with a negligible growth in the direction perpendicular to the nanosheets.^155^ Using a gas–liquid interface growth and heating the reactants at 60 °C, Xu et al. have prepared nanosheets of [Ni_3_(HITP)_2_].^156^
(d) **Electrochemical growth of thin films.** In this method, MOF thin films are formed on the surface of electrodes using electrochemical reactions. Depending on the type of redox reaction, oxidation or reduction, we can distinguish two types of deposition methods: anodic and cathodic. In the anodic deposition, the metal itself is used as the anode and is oxidized, with an applied voltage, to generate metal ions in the solution that react with the ligand. MOF particles start to grow on the surface of the anode. Ameloot and co-workers have prepared thin films of [Cu_2_(BTC)_3_] (HKUST-1) by immersing a copper electrode in a solution of H_3_BTC with methyltributylammonium methyl sulfate (MTBS) as the electrolyte using different applied voltages and times.^157^ In the cathodic deposition, both metal ions and ligands are present in the electrolyte solution. Ligand deprotonation occurs through electrochemical reactions, and the MOF particles are formed at the cathode surface. Dinca et al. have obtained thin films of Zn_4_O(BDC)_3_ (MOF-5) by this method.^158^ Hupp et al. have fabricated thin films of four different MOFs: NU-1000, UiO-66, HKUST-1, and MIL-53 by using an electrophoretic deposition method.^159^ In this case, the MOF thin films are prepared by using presynthesized MOF particles dispersed in a colloidal solution of the electrolyte. The MOF thin films grow on the surface of the electrode through electrostatic interactions.

## 5 Applications of Conducting MOFs

Conducting MOFs are well-known for several electrochemical and electronic applications in combination with the other established functionalities of porous MOFs. Here, we will discuss some examples of applications of conducting MOFs like charge storage^160^ (supercapacitors, Li-ion batteries, Li–S batteries, etc.), electrocatalysis^161−163^ (hydrogen evolution reaction, HER, oxygen evolution reaction, OER, oxygen reduction reaction, ORR, CO_2_ reduction reaction, CO_2_RR, etc.), sensing^164−166^ (gas sensing, organic molecule sensing, and biomolecule sensing), electronic devices, etc.

### 5.1 Charge Storage

Electrochemical charge storage has become a hot topic in order to obtain batteries with high charge storage densities, fast charging processes, large number of cycles, etc.^167^ Porous MOFs, with easily accessible metal sites along with large surface area, have become an interesting platform to study their charge storage properties.^168^ Dinca and co-workers have shown capacitance behavior of [Ni_3_(HITP)_2_] MOF by developing electrochemical double layers. The pristine material shows a capacitance of 18 μF cm^–2^ with excellent retention over 90% even after 10,000 cycles in TEABF_4_/ACN electrolyte. The authors have demonstrated that the presence of large pores within the MOF allows the diffusion of electrolytes, and this promotes the large recyclability over charging–discharging.^169^ Wang and co-workers have shown an excellent supercapacitor activity of [Cu_3_(HHTP)_2_] deposited on reduced graphene oxide. The authors prepared MOF nanowires on the substrate with a capacitance of 44.6 mF cm^–2^ at 5 mV s^–1^ with good recyclability and mechanical flexibility based on its large porosity and high electrical conductivity.^170^ Wechsler and co-workers have fabricated nanosheets of [Ni_3_(HIB)_2_] on Ni-foam. The obtained material shows a capacitance of 13.63 mF cm^–2^ at 0.1 mA cm^–2^ with 81% retention after 50,000 cycles.^171^ Bao and co-workers have reported high capacitance of Ni-HIB and Cu-HIB MOFs. Both redox-active frameworks show pseudocapacitance behavior with gyrometric capacitance values of 420 F g^–1^ and 215 F g^–1^ for the Ni-HIB and Cu-HIB MOFs, respectively, with a retention of around 90% after 12,000 cycles.^172^ Andikaey et al. have deposited thin films of NiCo-MOF on a glucose-modified liquid-phase exfoliated reduced graphene oxide substrate. The glucose moieties on the surface of the exfoliated graphene layers provide a large amount of donor hydroxide groups to coordinate the metal centers for the construction of MOFs on the substrate. The fabricated NiCo-MOF on the modified graphene layers shows excellent supercapacitor activity of 244 F g^–1^ with a specific capacitance of 4077.3 F g^–1^ at 2.5 A g^–1^ and a high charge density of 76.3 W h g^–1^.^173^ Jia and co-workers have deposited thin films of NiPc-MOF on Ni-foam to prepare a material with a specific capacitance of 22.1 mF cm^–2^ in an organic electrolyte and 11.5 mF cm^–2^ in water at 0.1 mA cm^–2^ with good recyclability and mechanical stability.^174^ Mohan et al. have developed a composite of a Ni/Co-MOF with reduced graphene oxide. The specific capacitance value increases from 978 F g^–1^ of the bare Ni/Co-MOF to 1162 F g^–1^ due to composite formation. The composite also shows high retention of capacitance after 5000 cycles.^175^ Jayaramulu and co-workers have made thin films of UiO-66 NH_2_ MOF on carboxylate-functionalized graphene through covalent attachment and have fabricated a device with a high capacitance of 651 F g^–1^ with excellent retention of 88% after 10,000 cycles of charging–discharging.^176^

Lithium-ion batteries (LIB) can store and release charge through the back and forth movement of Li^+^ ions between the anode and cathode.^177^ Li^+^ ions can be incorporated within the MOFs either on the metal nodes or loading it within the void space through ion exchange. Nishihara and co-workers have shown LIB application of the [Ni_3_(HITP)_2_] MOF based on the electron-carrying capacity of Li^+^ and PF_6_^–^ ions. This highly conductive MOF shows a high storage capacity of 115 mA h g^–1^ with a large specific energy density of 434 W h kg^1–^ at 10 mA g^–1^ with a retention of capacity after 300 cycles.^178^ Guo and co-workers have synthesized nanowires of Cu-CAT [Cu_3_(2,3,6,7,10,11-hexahydroxytriphenylene)_2_]. This conducting porous MOF shows LIB application based on Li^+^ ion diffusion. It shows a specific capacity of 631 mA h g^–1^ at 0.2 A g^–1^ with retention of ∼81% capacity after 500 cycles.^179^ Long et al. have shown LIB application of (H_2_NMe_2_)_2_Fe_2_(Cl_2_dhbp)_3_ and (H_2_NMe_2_)_2_Fe_3_(Cl_2_dhbp)_3_(SO_4_)_2_. The MOFs show specific capacities of 142 and 165 mA h g^–1^, respectively, in the presence of 0.1 M LiBF_4_ propylene electrolyte at 10 mA g^–1^ with recyclability.^180^ Yan et al. have reported LIB application of another MOF: Cu-HHTQ (HHTQ = 2,3,7,8,12,13-hexahydroxytricycloquinazoline) with a specific capacity of 657 mA h g^–1^ at 600 mA g^–1^ with a retention of capacity of ∼82% after 200 cycles.^181^ Finally, Wei et al. have deposited a fluorine-doped Co-MOF on reduced graphene oxide. This material shows a high lithium storage capacity of 1202 mA h g^–1^ at 0.1 A g^–1^ with good recyclability.^182^

Lithium–sulfur batteries rely on the charge storage between a sulfur cathode and a metallic Li anode.^183^ Porous conductive MOFs have gained much attention in this field based on their high charge transport properties, loading of sulfur/polysulfides within the void space, volume change during cycling, easy diffusion of electrolytes, reduced dissolution of polysulfides, etc. Zhao et al. have shown a large encapsulation of sulfur within Cu-BHT MOF through mutual interaction of guest sulfides with the metal centers of the framework and Li^+^ ions with S-donor sites of the ligand backbone. These authors propose (Li–S)_*n*_@Cu-BHT as a potential electrode for Li–S batteries.^184^ Cai and co-workers have shown Li–S battery application of porous conductive nanosheets of the 2D MOF [Ni_3_(HITP)_2_].^185^ The S-loaded framework: S@Ni-HITP was mixed with carbon nanotubes (CNTs) to prepare the cathode. Under the experimental condition, the cathode shows a high storage capacity of 1302.9 mA h g^–1^ with excellent capacity retention after 300 cycles, which is far better than S@CNTs or S@ZIF-67. The authors have suggested several reasons for such extraordinary performance, like high conductivity, mutual cooperation between MOF and CNTs, and porosity of the MOF to accommodate polysulfides. Wang and co-workers have reported the sulfur loading within the void space of Ni-HHTP MOF on self-supported carbon paper.^186^ The obtained material shows a high capacitance of 892 mA h g^–1^. Zhao et al. have reported Li–S battery applications of a composite of MIL-101(Cr) with graphene and S.^187^ The composite shows a high storage capacity of 809 mA h g^–1^ with 95% capacity retention after 134 cycles. Finally, Geng et al. have shown that the PPy-ZIF-67-S composite can act as a cathode in Li–S batteries. The composite shows a high specific capacitance of 1092.5 mA h g^–1^.^188^

### 5.2 Electrocatalysis

Electrocatalysis plays, and will continue to play, an important role in solving the major problems of fossil fuel consumption and environment related issues through electrochemical energy storage and conversion.^189^ High porosity and uniform distribution of catalytically active metal sites within the MOFs allow them to act as efficient electrocatalysts.^161−163^ However, their poor electrical conductivity and low chemical stability under the electrocatalytic conditions are the major problems for their electrocatalytic applications. The development of electrical conducting MOFs provides good opportunities for their electrocatalytic applications, though long-term stability is still a major concern. In this section, we will discuss some interesting examples of electrocatalytic applications of MOFs in hydrogen evolution reaction (HER), oxygen evolution reaction (OER), oxygen reduction reaction (ORR), and CO_2_ reduction reaction (CO_2_RR).

The hydrogen evolution reaction (HER) is a two-electron half-cell reaction in water splitting which occurs at the cathode over a wide pH range.^190^ Proton reduction takes place through a two-step process: (a) Volmer step, to produce a hydrogen intermediate from water or a proton by one-electron reduction and (b) Tafel or Heyrovsky step, to generate molecular hydrogen.^191^ Significant development has already been achieved by using transition-metal-based MOFs. Feng et al. have reported electrocatalytic HER activity of 2D nanosheets of Ni-TPHT (H_6_TPHT = 1,2,5,6,9,10-triphenylenehexathiol). The Ni-dithiolene moieties within the framework allow hydrogen evolution with an overpotential of 110 mV (10 mA cm^–2^ at 333 mV), which is far better than other doped-graphene materials.^192^ Marinescu et al. have reported electrocatalytic HER activity of two different MOFs with similar cobalt-dithiolene moieties: Co-BHT (H_6_BHT = 1,2,3,4,5,6-benzenehexathiol) and Co-TPHT (H_6_TPHT = 1,2,5,6,9,10-triphenylenehexathiol) which show substantial stability in acidic pH. At pH = 1.3, the overpotential values are 340 and 530 mV with a current density of 10 mA cm^–2^, for Co-BHT and Co-TPHT, respectively.^193^

The oxygen evolution reaction (OER), the anodic reaction of water splitting, is a four-electron oxidation process that is kinetically slower and more complex in nature than HER. Dubey and co-workers have reported OER activity of two different Co-pdc MOFs (pdc = 2,5-pyridinedicarboxylate) of different dimensionalities within the pH range 7–14. At neutral pH, the OER overpotentials are 0.77 and 0.46 mV for the 2D and 1D MOFs, respectively, for a current density of 1 mA cm^–2^. These values reduce to 0.36 and 0.25 mV at pH = 14.^194^ Yang et al. have reported OER activity of MIL-53(Fe)-2OH at an overpotential of 215 mV with a current density of 10 mA cm^–2^ in 1 M KOH.^195^ Tang et al. have shown electrocatalytic OER activity of ultrathin nanosheets of 2D NiCo-UMOFN at an overpotential of 180 mV with a current density of 10 mA cm^–2^ in 1 M KOH.^196^ Luo and co-workers have reported OER activity of CoCl_2_@Th-BPYDC framework (BPYDC = [2,2′-bipyridine]-5,5′-dicarboxylate) in acidic pH (0.1 M HClO_4_).^197^

Oxygen reduction reaction (ORR), the reverse cathodic reaction of OER, has significant importance in metal ion batteries and fuel cells.^198−200^ It may be a two- or four-electron process and can be conducted in both alkaline and acidic medium. Highly electrically conducting MOFs with M–N_*x*_ or M–O_*x*_ units are considered as potential ORR catalysts. Dinca and co-workers have reported the electrocatalytic ORR activity of the conducting [Ni_3_(HITP)_2_] MOF (HITP = 2,3,6,7,10,11-hexaiminotriphenylene) having Ni–N_4_ units on a glassy carbon electrode. The electrocatalytic onset potential is 0.82 V at a current density of 0.05 mA cm^–2^ in 0.1 M KOH solution.^201^ Yoon et al. have shown the four-electron electrocatalytic reduction of O_2_ by Co_0.27_Ni_0.73_-CAT (H_6_CAT = 2,3,6,7,10,11-hexahydroxytriphenylene) in a 0.1 M NaClO_4_ and 0.02 M PBS electrolyte solution. The catalytic cathodic peak appears at 0.41 V vs. reversible hydrogen electrode (RHE), which is much higher than the values of the corresponding monometallic Co-CAT or Ni-CAT MOFs.^202^ Mao et al. have reported the electrocatalytic ORR activity of two different MOFs: HKUST-1 and Cu-bipy-BTC (bipy-BTC = 2,2′-bipyridine-benzene-1,3,5-tricarboxylate).^203^

The CO_2_ reduction reaction (CO_2_RR) to value-added chemicals like CO, HCOOH, CH_4_, CH_3_OH, etc., is highly desirable to develop another alternative and sustainable source of energy and reduce CO_2_ emissions.^204^ The high chemical stability of the CO_2_ molecule is the main challenge in this technology. It is considered that CO_2_RR proceeds through three different steps: (a) binding of CO_2_ to the active sites, (b) reduction of CO_2_ via either electron transfer or H^+^ attachment to the CO_2_ molecule, and (c) rearrangement and detachment of the product from the catalyst.^205^ Different conducting MOFs such as copper-based MOFs and MOFs containing porphyrin-based organic moieties have been studied for electrochemical CO_2_ reduction. Gu et al. have reported the electrocatalytic CO_2_ reduction by a porphyrin-based MOF [Cu_2_(CuTCPP)] (CuTCPP = 5,10,15,20-tetrakis(4-carboxyphenyl)porphyrin-Cu(II)). The MOF shows CO_2_RR at an overpotential of −1.55 V vs. Ag/Ag^+^ to convert CO_2_ to formate with a faradaic efficiency (FE) of 68.4%.^206^ Kumar et al. have reported the electrocatalytic CO_2_ reduction by HKUST-1 deposited on a glassy carbon electrode with formation of oxalic acid as the major product. The MOF-coated glassy carbon electrode shows electrocatalytic CO_2_RR activity at an overpotential of −1.12 V vs. Ag/Ag^+^ with 51% FE.^207^ Hod et al. have shown the electrocatalytic CO_2_ reduction of a MOF-525 containing Zr_6_ metal nodes and Fe-TCPP as the building blocks. The electrocatalysis was carried out by depositing thin films of MOF-525 onto FTO substrates. These thin films show electrocatalytic CO_2_ reduction to CO at an overpotential of −1.3 V vs NHE with 100% FE in the presence of trifluoroethanol.^208^ Bao et al. have studied the electrocatalytic CO_2_ reduction by a series of MOFs with similar sodalite topology: ZIF-7, ZIF-8, and ZIF-108. ZIF-8 shows CO_2_ reduction at an overpotential of −1.1 V vs. RHE to CO with 81% FE, while ZIF-108 shows a similar behavior at an overpotential of −1.3 V vs. RHE.^209^

### 5.3 Electrochemical Sensors

Highly selective and sensitive electrochemical sensing has several important applications, like environmental monitoring, disease diagnosis, rare element detection, and so on. In an electrochemical sensing device, the analyte interacts with the surface of the sensor that produces an electrical signal in response to the analyte–sensor interaction.^210^ Conductive MOFs are used as potential electrochemical sensors based on their excellent properties such as high porosity, tunable functionalization, reproducibility, efficient charge transfer properties, etc.^164,165,211^ In some cases, MOFs are also assembled with carbon paste electrodes (CPE) and glassy carbon electrodes (GCE) to implement them in electrochemical sensing devices. To date, different types of electrochemical sensing methods, such as chemresistive, chemcapacitive, impedance, Kelvin probe, and field effect transistors, have been developed to detect different types of analytes.^212^ In the next sections, we will briefly discuss the electrochemical sensing properties of different MOFs and MOF-composites by classifying analytes into four categories: gases, ions, molecules, and biomolecules.

#### Gas Sensing

Dinca group have reported the first example of chemresistive sensing of ammonia gas using 2D conductive [Cu_3_(HITP)_2_] within the detection limit 0.5–10 ppm. The interaction between ammonia and copper atoms within the MOF is the underlying reason for such selective sensing of ammonia.^213^ These authors have also developed a cross-reactive chemresistive sensor array using Cu_3_(HHTP)_2_, Cu_3_(HITP)_2_, and Ni_3_(HITP)_2_ to detect and classify 16 organic compounds based on their functional groups: alcohol, ether, ketone, aromatic, amine, and aliphatic.^88^ Xu et al. have fabricated thin films of [Cu_3_(HHTP)_2_] by the spray layer by layer method that show highly selective detection of ammonia in the range 1–100 ppm.^214^ Mirica et al. have shown highly selective detection of NO and H_2_S (with detection limits of 0.16 and 0.23 ppm, respectively) by self-organizing [Ni_3_(HHTP)_2_] on textiles.^215^ Dmello and co-workers have reported the chemresistive sensing of SO_2_ by a UiO-66-NH_2_ MOF in Ar atmosphere.^216^ Hupp et al. have reported the sensing of H_2_ (5%) gas by SnO_2_@NU-1000 MOF.^117^ Long et al. have shown that the electrical conductivity of {Cu[Ni(pdt)_2_]} can be altered by selective adsorption of gaseous hydrocarbons such as ethane, ethylene, acetone, propane, and *cis*-2-butene at room temperature.^217^ Kitagawa et al. have demonstrated the sensing performance of [Cu_3_(HHTP)(THQ)] toward ammonia through IR and PXRD analysis.^218^

#### Ion Sensing

Zhang et al. have developed an electrochemical sensing device with [Cu_3_(BTB)_2_(H_2_O)_3_] on a carbon paste electrode which was further modified by the absorption of hexacyanoferrate(III) ions. The obtained [Fe(CN)_6_]^3–^/[Cu_3_(BTB)_2_(H_2_O)_3_]/CPE device shows selective detection of nitrite ions with a detection limit of 40 nM.^219^ Roushani et al. have shown the electrochemical sensing of Cd^2+^ ions by a TMU-16-NH_2_ modified electrode.^220^ Mirica et al. have reported the potentiometric detection of K^+^ and NO_3_^–^ ions by integrating the 2D conductive MOFs [Ni_3_(HHTP)_2_], [Cu_3_(HHTP)_2_], and [Co_3_(HHTP)_2_] into an ion-selective membrane-coated solid-state potentiometric device. The detection limits are 0.631(1) μM and 0.501(1) μM for K^+^ and NO_3_^–^ ions, respectively.^221^

#### Molecule Sensing

Zhang et al. have reported chemresistive sensing of moisture by porous ZIF-67 MOF with a detection limit of 5 ppm.^222^ The same group also reported the chemresistive sensing of trimethylamine by [Co(im)_2_]_*n*_ MOF with a detection limit of 2 ppm.^223^ Zhan et al. have reported the selective detection of H_2_O_2_ by ZnO@ZIF-8 nanostructures deposited on a FTO substrate.^224^ Hosseini et al. have fabricated an electrochemical sensing device by immobilizing Au-SH-SiO_2_ nanoparticles on HKUST-1 that can detect and electrocatalytically oxidize hydrazine with a detection limit of 10^–8^ M.^225^

#### Biomolecule Sensing

Li et al. have developed an electrode material by depositing Ag nanoparticles on MIL-101(Fe) on modified glassy carbon electrode which shows selective detection of tryptophan. The modified electrode presents a higher oxidation current in the presence of tryptophan, which increases with the amount of tryptophan, with a detection limit of 140 μM.^226^ Deep et al. have developed a thin film of the MOF Cd-ATC (ATC = 2-aminoterephthalate) on ITO for pesticide detection. The thin film was modified with antiparathion antibody and then used for the detection of parathion with a detection range of 0.1–20 ng mL^–1^).^227^

### 5.4 Electronic Devices

Prasoon et al. have prepared a TCNQ-doped MOF thin film with diode-like behavior. The thin films were prepared by depositing Cu-BTEC (BTEC = 1,2,4,5-benzenetetracarboxylate) on self-assembled multilayers (SAM) with the layer by layer (LBL) technique. The films were doped with TCNQ molecules through coordination of the cyano-groups of TCNQ with the metal sites of the framework. I–V measurement shows that the TCNQ@Cu-BTEC thin films act as a Schottky diode with 10^5^ rectification ratio.^228^ Wu et al. have developed a porous field-effect transistor (FET) based on thin films of the highly conducting [Ni_3_(HITP)_2_] MOF prepared using the gas–liquid interfacial method. The obtained thin film was deposited on a SiO_2_ substrate by the stamp method, and then Au electrodes were patterned on the surface of the MOF film to develop the FET device. This FET device shows p-type behavior with an on/off ratio of 2 × 10^3^ and field effect mobility of 48.6 cm^2^ V^–1^ s^–1^.^229^ Wang et al. have developed a FET device using the same [Ni_3_(HITP)_2_] MOF on a Si/SiO_2_ substrate using in situ solid–liquid interfacial method. The FET device shows an on/off ratio of 2.29 × 10^3^ with a mobility of 45.4 cm^2^ V^–1^ s^–1^.^230^

## 6 Challenges and Perspectives

After decades of intense research, the field of conducting MOFs has accomplished important results and has shown that electrical conducting MOFs may act as functional materials in fields such as charge storage, electrocatalysis, electrochemical sensing, etc. However, one of the major challenges for their industrial application is their nonscalable synthesis and high cost, in most cases. Moreover, their limited stability under experimental conditions also hinders their use. Therefore, there are still several challenges to be faced for their industrial development:

(a) **High electrical conductivity.** There are still very few MOFs reported with high electrical conductivities (>1 S cm^–1^), but most electronic applications of MOFs require high conductivity. Therefore, there are still much effort to be done to develop highly conducting MOFs. Most of the highly conducting MOFs are obtained using planar organic S- or N-donor ligands since they present a high metal–ligand orbital overlap. Therefore, research efforts should be carried out to find out adequate metal–donor atom overlaps for better conductivity using other donor atoms such as O, Se or P and other transition metal atoms. Radical-based ligands also provide opportunities to develop highly conducting MOFs. Besides anilate-derivative ligands, other redox-active ligands should be used to design new conducting MOFs, especially those forming stable organic radicals.
(b) **MOF-composites interactions.** The synthesis of MOF-composites with synergetic interactions between MOFs and conducting materials is a second important challenge to be faced. Unfortunately, in most cases, the composites are formed by simply mixing the MOFs with the conducting matrices. This fact leads to nonuniformity of the material with weak interaction between the MOFs and the conducting material, limiting their stability. Furthermore, the weak interactions between the MOF and the conducting matrix also limits the charge transport. A possible way to overcome these low interactions may be the direct synthesis of the MOFs as nanoparticles inside the functionalized conducting matrices.
(c) **Stability.** One of the major challenges in MOF chemistry (not only in conducting MOFs), is their limited stability under experimental conditions. Most highly conducting 2D MOFs contain metal centers with square planar coordination environments that make them highly suitable for electrocatalytic applications but reduce their stability due to potential solvent coordination to the axial vacant sites. The design of conducting MOFs with strong metal–ligand coordination bonds is a good strategy to increase their stability.
(d) **Transport Mechanisms analysis.** In only a few cases, the charge transport mechanism has been established including both intrinsically and extrinsically conducting MOFs. The charge carrier types (holes or electron), charge carrier density, and their mobility are also important parameters to characterize conducting MOFs. Therefore, mechanistic studies need to be performed in order to design suitable MOFs for different applications. A combination of experimental and theoretical investigations may help to establish the detailed charge transport mechanisms in conducting MOFs.
(e) **Applications.** For charge-storage MOFs, it is highly desirable to correlate the charge storage with the active sites of the MOFs. The uptake/release of metal ions through the channels of the MOFs is a key factor in metal-ion batteries. These phenomena should be studied in detail in order to develop MOFs with a high storage capacity and charge–discharge recyclability. Electrocatalysis (HER, OER, ORR, and CO_2_RR) is one of the most important applications of conducting MOFs. However, their performances and stability are still far behind noble metal catalysts. Further investigations are required to improve the electrocatalytic performance of conducting MOFs. The use of conducting MOFs with two (or more) different metal ions may be an appropriate strategy to increase the catalytic performance, as already observed in zeolites and other heterogeneous catalysts. Finally, electrochemical sensors based on conducting MOFs require the presence of strong interactions between the sensor and the analyte. A possible way to increase these interactions may be the use of ligands with many functional groups. The use of conducting MOFs with different metal ions and ligands simultaneously is expected to increase the selectivity of these sensors.

## 7 Conclusions

The synthesis of conducting MOFs has become a very active area in recent years since the addition of electrical conductivity to these multifunctional materials may lead to applications in fields such as electrochemical sensors, photovoltaic devices, electrocatalysis, batteries, etc. The intrinsic hybrid tunable structures of most MOFs and their capacity to insert many different types of guests have led to the preparation of an increasing number of conducting MOFs and, more importantly, to the rational improvement of the conductivity in some of these examples.

However, most of these conducting MOFs show low room-temperature conductivities and semiconducting behaviors, which largely limits their use in the aforementioned fields. Luckily, as we have shown here, the work of many qualified and active research groups is changing this trend by developing different strategies to increase the electrical conductivity in MOFs. The first results show that this is, indeed, possible, as shown by some synthesized MOFs with high conductivities and even with metallic behavior.

For the intrinsically conducting MOFs (those where the electrical conductivity takes place through the framework of the MOF), these strategies can be summarized in (i) the use of mixed-valence metal ions and/or ligands, which favor the electron transport, (ii) the use of soft donor-atom-based ligands that lead to better metal–ligand orbital overlap, favoring the through-bond electron transport, and (iii) the use of planar and highly conjugated ligands to favor the interchain/interlayer π–π interactions, favoring the through-space charge transport.

In the case of the extrinsically conducting MOFs (those where the electrical conductivity is achieved by inserting electroactive guests species that facilitate the electron transfer), the strategies to improve conductivity can be summarized in (i) the insertion of metal ions and/or metal nanoparticles that may create a pathway for the electron transfer, (ii) the insertion of electroactive molecules (either donors or acceptors) that may change the electron density in the framework and facilitate the electron transfer (either through the framework or through the guest), and (iii) the incorporation of well-known organic conducting polymers in channels or in the interlayer space of the host MOFs, to create a conducting pathway inside the MOF. In most cases, the insertion of the guests can be done either postsynthetically or by in situ template-assisted synthesis. In the case of the organic conducting polymers, the insertion of monomers and their posterior polymerization constitutes a very useful strategy.

## ABBREVIATIONS

MOF: Metal–organic framework
CP: coordination polymer
BEDT-TTF: bis(ethylenedithio)-tetrathiofulvalene
pdt: 2-3-pyrazinedithiolate
H_2_dhbq: 2,5-dihydroxy-benzoquinone
Cl_2_dhbq: 2,5-dichloro-3,6-dihydroxo-1,4-benzoquinone
H_6_HHB: hexahydroxybenzene
H_6_HHTP: 2,3,6,7,10,11-hexahydroxytriphenylene
DOBDC: 2,5-dioxidobenzene-1,4-dicarboxylate
H_4_DSBDC: 2,5-disulfhydrylbenzene-1,4-dicarboxylic acid
H_6_HIB: hexaiminobenzene
HITP: hexaiminotriphenylene
OIPc: octaiminophthalocyanine
BHT: benzenehexathiolate
IR: infrared
XPS: X-ray photoelectron spectroscopy
NPs: nanoparticles
BDT: benzenedithiolate
BTT: benzenetetrathiolate
TPHT: triphenylenehexathiolate
PcFe-O_8_: (2,3,9,10,16,17,23,24-octahydroxyphthalocyaninato)Fe
H_6_AT: 1,3,5-triaminobenzene-2,4,6-trithiol
IT: 1,3,5-triiminobenzene-2,4,6-trithiolate
3,5-Dmpip-dtc: 3,5-dimethylpiperidine dithiocarbamate
DCTP: 4′-(3–5-dicarboxyphenyl)-4,2′:6′,4″-terpyridine
H_2_BDT: 5,5′-(1,4-phenylene)bis(1H-tetrazole)
tri: triazolate
TTFTB: tetrathiafulvalene-tetrabenzoate
4-Cl-BA: 4-chlorobenzoate
4,4′-bipy: 4,4′-bipyridine
ndc: 2,6-napthalenedicarboxylate
1,10-phen: 1,10-phenanthroline
H_2_ABEDBA: 4,4′-(anthracene-9,10-diylbis(ethyne-2,1-diyl))dibenzoic acid
DPNDI: *N,N*′-di(4-pyridyl)-1,4,5,8-naphthalenetetracarboxdiimide
H_2_OBA: 4,4′-oxybisbenzoic acid
TCNQ: 7,7,8,8-tetracyanoquinodimethane
TTF: tetrathiafulvalene
PEDOT: poly(3,4-ethylenedioxythiophene)
PANI: polyaniline
PPy: polypyrrole
amd: bis(N,N′-di-*i*-propylacetamidinato
NU: Northwestern University
PSM: postsynthetic modification
BEDT-TSF: bis(ethylenedithio)-tetraselanafulvalene
H_3_BTC: benzene-1,3,5-tricarboxylic acid
HKUST: Hong Kong University of Science and Technology
XRPD: X-ray powder diffraction
TEM: tunnelling electron microscope
SEM: scanning electron microscope
H_2_TPyP: 5,10,15,20-tetra-4-pyridyl-21H,23H-porphine
d,l-lac: lactate
pybz: 4-pyridine benzoate anions
H_4_TBAPy: 1,3,6,8-tetrakis(*p*-benzoic acid)
NiCB: [Ni(dicarbollide)_2_]
IDE: interdigitated electrodes
BPDPNDI: N,*N*′-bis(4-pyridyl)-2,6-dipyrrolidyl naphthalenediimide
TCPB: 1,2,4,5-tetrakis(4-carboxy phenyl)benzene
DFDNB: 1,5-difluoro-2,4-dinitrobenzene
DNT: dinitrotoluene
MV^2+^: methyl viologen
NIR: near infrared
CT: charge transfer
UiO: University of Oslo
EPR: electron paramagnetic resonance
MALDI: Matrix-Assisted Laser Desorption/Ionization
EP: electro-polymerization
H_2_NDC: 2,6-napthalenedicarboxylicacid
HPCA: 4-pyridinecarboxylic acid
FTO: fluorinated tin oxide
MIL: Matériaux de l′Institut Lavoisier
SAM: self-assembled monolayers
H_2_BDC: benzene-1,4-dicarboxylic acid
MTBS: methyltributylammonium methyl sulfate
HER: hydrogen evolution reaction
OER: oxygen evolution reaction
ORR: oxygen reduction reaction
CO_2_RR: CO_2_ reduction reaction
LIB: lithium ion battery
HHTQ: 2,3,7,8,12,13-hexahydroxytricycloquinazoline
CAT: 2,3,6,7,10,11-hexahydroxytriphenylene
H_2_BPYDC: [2,2′-bipyridine]-5,5′-dicarboxylic acid
Cu-TCPP: 5,10,15,20-tetrakis(4-carboxyphenyl)porphyrin-Cu(II)
FE: Faradaic Efficiency
RHE: reversible hydrogen electrode
CPE: carbon paste electrodes
GCE: glassy carbon electrodes
H_3_BTB: 4,4′,4″-benzene-1,3,5-triyl-tribenzoic acid
TMU: Tarbiat Modares University
Im: imidazolate
H_2_ATC: 2-aminoterephthalic acid
H_4_BTEC: 1,2,4,5-benzenetetracarboxylic acid
